# Conical Intersections and Slopes Nearby, Inferred From Coherent Oscillations and From Photoproducts

**DOI:** 10.1002/cphc.70617

**Published:** 2026-11-12

**Authors:** Werner Fuß

**Affiliations:** ^1^ Retired From Max‐Planck‐Institut für Quantenoptik Garching Germany

**Keywords:** chemistry, conical intersection, diffraction, intramolecular force, isomerization, photodissociation, singlet state, thermalization, wave packet

## Abstract

Conical intersections of potential surfaces control photochemical reactions. There has been only limited success to determine their structures in ultrafast spectroscopy, including time‐resolved diffraction, despite recent progresses. It is here pointed out that directions of slopes on potentials can sometimes be easily recognized from coherent oscillations. They not only help to find conical intersections but sometimes play a central role, e.g., for controlling a wavelength dependence of a photoreaction. Slopes on potentials are forces. The idea that slopes in the Franck–Condon region often precede reactive forces (e.g., those of pericyclic reactions) rationalizes a number of empirical rules with wide application, especially if combined with the idea that a slight distortion makes some coordinates Franck–Condon active. Examples considered are cis‐trans isomerization (with competing reactions) of polyenes and of alkenes including ethylene, ring opening of cyclohexadiene and cyclobutenes, intramolecular charge transfer (dimethylamino‐benzonitrile), and photodissociation of metal carbonyls. Based on paths and their intersections, a solution is also suggested for the conflict between ultrafast singlet and triplet channels in metal carbonyls. In times below a few picoseconds, all processes are coherent, there is no thermalization and no vertical relaxation, and the wave packet stays compact all along the path.

## Graphical abstract

The vibrational coordinate of the observed oscillation indicates the direction of the slope down from the conical intersection. Slopes are forces that drive the photochemical reaction.

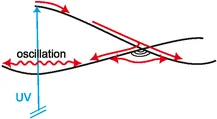

## 1 Introduction

Compared with the early times of photochemistry, when transitions between excited and ground states were rationalized by matrix elements and densities of states, consideration of the paths on potential surfaces (e.g., in the book of Klessinger and Michl [1]) provided much more insight [2]. A jump forward in understanding was made by progress of quantum chemistry in the early 1990s, when theorists revealed the ubiquity of conical intersections (CIs or CoIns) between the potential surfaces. Pioneering calculations in organic photochemistry were made by the group of Robb, Olivucci, Garavelli, and Bernardi. Their brief review [3] on pericyclic reactions and polyene cis‐trans isomerization may serve as an illustration: From the initially excited (“spectroscopic”) state (1B), the wave packet (i.e., the molecule) goes down via a first (1B/2A) CoIn to an optically dark two‐electron excited state (2A) and reaches its minimum, which is displaced and acts as a collection well. The outlet from there is the (2A/1A) S_1_/S_0_ CoIn. The collection basin plus upper part of the double cone act like a funnel, whereas on the lower cone the path branches: The slopes accelerate the wave packet either back to the reactant (resulting in internal conversion) or to the product(s).

A useful collection of articles is compiled in the two books on conical intersection by Domcke, Yarkony, and Köppel [4, 5].

Actually, the intersection is not a point but an intersection space (or intersection seam, IS) with dimensions *f *– 2, where *f* is the number of internal degrees of freedom. Two remaining coordinates span the branching space; moving in their direction lifts the degeneracy (or in the opposite direction generates it). Usually, one takes for them the directions, where the energy difference of the potentials increases fastest (gradient difference vector) and where the vibronic interaction grows fastest (nonadiabatic coupling (NADC) vector or derivative coupling vector). By CoIn one usually means a minimum in the IS (minimum energy conical intersection, MECI) or a region nearby. There can be several such minima, often corresponding to different reactions.

Obviously, the direction of displacement of the last CoIn and of the slopes nearby play a central role in guiding the molecule to the product(s) [3]. These directions have been found out primarily by quantum chemical calculations. But in spite of much progress, this task is sometimes still a challenge. Experimental verification is therefore desirable and is a goal of continuing interest. On the other hand, experiments—including time‐resolved diffraction—are usually interpreted on the basis of calculations.

There is general agreement that ultrafast (<1 ps) passing from a higher to a lower potential indicates crossing via a CoIn, which is easily accessible, that is without activation energy. Beyond such a proof of existence, various spectroscopic methods have been tried to find more electronic (also structural) details, and diffraction methods are being developed to pin down the geometrical structures and their evolution. Two examples may be representative:

1. Time‐resolved X‐ray absorption spectroscopy of ethylene [6], after ππ* excitation, revealed after some delay a split 1s → π transition. This indicates two nonequivalent C atoms, just as expected with the one‐sided pyramidalization that is predicted by quantum chemistry for ethylene after leaving from its perpendicular minimum (see, e.g., [1]). Because the authors’ calculations indicate this geometry also for the last (S_1_/S_0_) CoIn, they attribute this signal to the conical intersection. On the other hand, we argued that the molecule crosses over to the ground state in a region of the IS that involves in addition a partial H migration [7]. This view is underlined in Section 4.1.
2. Ultrafast electron or X‐ray diffraction has recently achieved temporal and spatial resolutions (160  fs or below 100 fs, sub‐Ångström) and sensitivity also for light elements such as carbon, a progress which enabled the time‐resolved mapping of interatomic distances in ring‐opening of 1,3‐cyclohexadiene and the subsequent conformer isomerization in the hot hexatriene product [8, 9, 10]. The results fully agree with the path (including the changes of direction) predicted in the accompanying time‐dependent quantum‐chemical calculation. This is very encouraging, of course, and further progress may be hoped for. As expected for a diffraction method, information on the involved states and CoIns is at best very indirect. By contrast, a previous time‐resolved spectroscopy of the same process [11] revealed the states (Section 5.2) and, probably due to the better time resolution (13 fs), that the Woodward–Hoffmann rules are switched on only with delay (Section 5.4) and that the molecular symmetry is broken, beginning near the first (S_2_/S_1_, 1B/2A) conical intersection (Section 5.2); this experiment agreed with the calculated path, too.

Both examples also demonstrate that the interpretation of time‐resolved spectroscopy or diffraction very much profits from quantum chemistry. On the other hand, because such calculations keep being further developed, experiments can help to check them.

A review of 2017 by Ischenko, Weber and Miller [12] on transient structure in reaction dynamics compiles information primarily from diffraction experiments but also from ultrafast spectroscopy and quantum chemistry. It also covers two of the systems discussed below, cyclohexadiene ring opening (Section 5) and photodissociation of iron pentacarbonyl (Section 7).

A general difficulty is that a CoIn is no stationary point, and passing through it only takes a fraction of a vibrational period [13]. The hope was, its localization would give information on the direction of the reaction. However, it is also the slopes in the branching space, which point to the reaction products (together with momentum possibly accumulated on the preceding path). So, why not take from the products information on such slopes and the direction of displacement of the CoIns? Slopes can also stimulate coherent oscillations that are sometimes observed in time‐resolved experiments. If they can be assigned to known molecular vibrations, they also give information on the direction of slopes, often on potentials higher than the ground state. Hence, slopes seem less elusive than CoIns.

Such sources of information have not received widespread attention, although it was the structure of the photoproduct, which was the first experimental support of a calculated CoIn geometry (Hula twist, Section 3) and the oscillations in DMABN, which pointed to the (still controversial) slope directions and the S_2_/S_1_ CoIn (Section 6.1). Therefore, this Perspective collects a number of cases with such evidence, mainly from published work of our own. The examples are also used to comment on more recent calculations and experiments.

Pointing to the importance of slopes also means that one should not only try to find out the geometry of CoIns but also pay attention to their surroundings.

The experimental evidence for slopes is incomplete: It only provides components of the slope vectors and information only on directions. Furthermore, not every slope gives rise to an observable coherent oscillation, and photoproducts do not reflect temporary deformations (Section 2.1). By contrast, calculations of potentials and of trajectories routinely determine the local gradients. It is desirable to directly compare the two sources of information.

It is also pointed out once more [2, 14] that down‐slopes on the potentials are driving forces, a concept familiar to chemists that may also stimulate fruitful interpretations.

The present contribution also wants to stimulate questions and interpretations, at the risk of speculations. That is, not only to find out (by ultrafast spectroscopy or diffraction or by calculations) *that* something happens but also why and how.

## 2 Introduction 2: Further Properties of CoIns and Some Remarks on Time‐Resolve Mass‐Spectroscopy With Multiphoton Ionization

### 2.1 CoIns

In a recent review [15], Boeije and Olivucci classify CoIns and ISs in organic photochemistry, giving many examples. In particular, they systematically consider cases where several pathways are possible and identify factors responsible for selection between them. Such questions are also relevant in the following sections. They also draw information on the participating coordinates from coherent oscillations, although in a slightly different way than done here: They consider in detail phase effects in vibrational coherences, in particular in rhodopsin and similar compounds. The relative phase of two such coherent oscillations (torsion and pyramidalization in the case of the chromophore of rhodopsin) determines the direction of passing through a CoIn or the exact location in the IS, hit by the wave packet. This in turn influences the quantum yield of cis‐trans isomerization. In our work, we primarily consider the direction of wave packet momentum, resulting from (varying) directions of slopes.

Some additional remarks on CoIns may also be helpful for the following:

• As said in the previous section, an ultrashort relaxation time indicates a path through a CoIn. Some quantum chemical works (on cyclohexadiene and metal carbonyls, mentioned in Sections 5.5 and 7 near the end of comment 4) indicate vertical S_*n *_→ S_1_ relaxation (radiationless transition) before the photochemical reaction from S_1_. But no vertical relaxation is ultrafast. We made a strong point for this statement in comparing the S_1_ lifetime of benzene (100 ns without excess energy) with the more than six orders of magnitude shorter ones of cyclopentadiene and cycloheptatriene [16]. In the latter two molecules, photochemical H migration (i.e., a nonvertical process) provides easily accessible CoIns, whereas the formation of prefulvene from benzene requires an activation energy [2, 16]. By contrast, densities of states and S_1_/S_0_ matrix elements (which would be used in vertical processes) would be similar for all three molecules [16]. Even in the nanosecond process in benzene, the CoIn mechanism provides more insight [2]. Another example is ethylene, where radiationless relaxation from the perpendicular minimum along a vertical path was calculated to take 1 ns, whereas the measured leaving time (obviously through a CoIn) is 17 fs [7] (Section 4.1).
• Potentials near CoIns are often drawn like double cones with steep slopes both, below and above the apex. In fact, this is correct in first order in the immediate surrounding of the apex. Higher orders often cause a flattening at higher energies (examples: ethylene and cyclohexadiene, Sections 4 and 5). If the upper cone is also steep, an incoming wave packet is usually deflected and circulates to some extent in this funnel; in this way, memory is lost, where it came from. However, if the potentials are very flat above a CoIn (Figure 1) and if in addition, there is not much angle dependence (if there is no valley in the entrance), the wave packet does not change its direction. So, a flat CoIn entrance with not much angle dependence seems a prerequisite for a memory of direction. This is how some wavelength dependences are interpreted below (Section 4.3, alkenes; Section 4.3.2, cyclobutenes).

**FIGURE 1:**
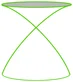
A conical intersection (CoIn), in which the upper cone is flatter than the lower one. The flatness and an additional absence of a valley in the upper cone infer that the direction of an incoming wave packet is not changed. If also the upper cone is steep, a tangentially incoming wave packet (whose trajectory would not point directly to the apex) first circulates, so that information on the direction is also lost.

• It should usually be asked: What interaction brings the upper potential down and the lower potential up toward the CoIn? For example, the (π antibonding) ππ* state of alkenes lowers its energy by C=C twisting, until a minimum is reached at 90° torsion. There, a two‐electron excited state takes over. (It results from correlation diagrams [1]). Because this state is zwitterionic [1], its energy is further lowered by pyramidalization (rehybridization toward sp^3^) of one C atom; a partial H migration also contributes (see Section 4.1). However, in polyenes, the two‐electron excited state is not zwitterionic [1]. But an attractive 1,3‐intraction helps (the C_2_ atom is pyranidalized, too); it can initiate Hula twist (Section 3). In the discussion on Hula twist versus one‐bond flip, the defendants of the latter usually do not ask: How to lower the energy further from the initial perpendicular minimum?
• Instead of one MECI (“CoIn”), theorists sometimes calculate a line of low‐lying CoIns, a minimum‐energy path (MEP) in the IS. Points of higher energy on this line may be reached only, if the molecule is excited by sufficiently energetic photons. Some wavelength dependences may have such an origin; examples are suggested for cyclohexadiene ring opening (Section 5.5) or the increased importance of sigmatropic H shift at shorter wavelengths in alkenes (Section 4.3). It should be kept in mind that in times below one or a few picoseconds, the molecule cannot search around to find the lowest‐energy region or even thermally equilibrate. The wave packet may hence deviate from a minimum energy path. Absence of equilibration is clearly demonstrated by the coherence of the oscillations with dephasing times of 1 ps or slightly longer. Coherent motion of the wave packet and hence the absence of thermalization is actually a prerequisite for wavelength dependences of the two kinds discussed above, i.e., those which depend on energy or on the direction of the trajectory. This same prerequisite is also required for the explanation [17] how a predistortion of a molecule controls the outcome of many photochemical reactions (Section 5.4.1).

Calculations indicated that the MEP in the IS is nearly parallel to the MEP on the excited potential (“the reaction path”) in 1,3‐cyclohexadiene [18], in DMABN [19] and in more examples [20], so that one can ask, how the molecule finds the crossing region. We suggested (and presented evidence) that cyclohexadiene is accelerated at the preceding CoIn in the right direction (Section 5.2) and in DMABN there is an early driving force toward a twist and that the CoIn is twisted (Sections 6.1 and 6.3).

• Some of the geometrical distortions at a CoIn (and directions of slopes) may be only temporary and leave no trace in the photochemical products. Examples are bond‐length changes or the one‐sided pyramidalization in ethylene. So, information from photoproducts is incomplete. The same is true for the coherent oscillation for an additional reason: Their observability depends on the detection method. That is, there is always a need for supplementary experiments, including also diffraction methods.
• For further properties of CoIns and their properties, see the review of Boeije and Olivucci [15] or the Klessinger–Michl book [1].

### 2.2 Time‐Resolved Multiphoton Ionization With Mass‐Selective Detection

Most of our time‐resolved work was done in the gas phase, using multiphoton ionization (in most cases using 800 nm for the probe) with mass selective detection. One could also call it time‐resolved mass spectroscopy, although we did not always monitor all fragment ions. It has a number of advantages over other spectroscopies. Nevertheless, sometimes doubts were expressed concerning our short time constants and time resolution or our assignments or the many constants that were extracted. Some comments seem therefore appropriate.

• A number of our time constants with assignments are in the meantime confirmed by independent methods, for example, the early ones in ring opening of 1,3‐cyclohexadiene by time‐resolved photoelectron spectroscopy (see the review [21]). Many more confirmations are reported below.
• At a time before we shortened our pulses from 100 to 10 fs, we extracted time constants in the 10 fs range by deconvolution. Also, two consecutive short times can be extracted, using independent mass signals; this is analogous as one can separate two stars with distance below the optical resolution, if their emission spectrum differs, as explained in [22]. But deconvolution could be problematic during the pump‐probe overlapping time: A nonlinear interaction between pump and probe might occur in addition to the (nonlinear) multiphoton probe absorption. In fact, we have observed such a case (see [22]), where we also explain how to recognize and avoid such problems. However, in general, when we repeated some experiments with the shorter pulses, the time constants were confirmed (e.g., in Cr(CO)_6_ [22] or cyclohexadiene [11]). But one should note that fast oscillations cannot be extracted by deconvolution [22]. For them, one needs the short pulses.
• The many independent mass signals helped to extract the many time constants and oscillation frequencies. A given constant was always contained in several signals, though with different weights.
• In the first few picoseconds the wave packets move coherently, as demonstrated by the observed coherent oscillations. One could expect also some initial time of acceleration of the wave packets (“latency time”), as shown in some calculations. Also the rest of the decay is not really exponential in trajectory calculations. Nevertheless, we observed (multi)exponential behavior from the beginning (although often modulated by coherent oscillations, resulting in exponentials multiplied by periodic functions). We tentatively interpreted it by the fact that the observation windows extend over some energy range so that some coherent effects may be averaged out. Therefore, we also prefer “traveling time” over “lifetime” for the time constants. (The concept of traveling times is nicely illustrated with Cr(CO)_6_, where different excitation wavelengths led to different path lengths [22]).
• A great advantage is that multiphoton ionization can be monitored over all states (including optically dark states), from the Franck–Condon region down to the hot ground state. States of (strongly) different energy can be distinguished by the order of multiphoton ionization (which is even easier with longer wavelengths, see the last point). By contrast, transient absorption can typically monitor only one state. Transient photoelectron spectroscopy using near‐UV photons cannot detect low‐lying states; if photons of ≥20 eV (e.g., from high harmonics) are used, the ground state can also be detected, but the signal‐to‐noise ratio still needs improvement. The difficulty of assigning absorption spectra and photoelectron spectra of molecules far from equilibrium geometry is often underestimated; spectra from different states usually overlap.
• From the Franck–Condon region, fragmentation was often negligible; that is, only the molecular ion was observed. The next observation region often showed no molecular ion anymore. Fragmentation was strongest (e.g., down to H^+^) from the hot ground state (of reactant or product). In this way, it was easy to distinguish it from unexcited molecules. By contrast, transient absorption or photoelectron spectroscopy requires subtraction of a background, the spectra of unexcited molecules. In transient diffraction, the ground‐state signals (pair distribution functions) must even be subtracted from those of all observation windows.
• Because for ionization there is no dark region, there is also no gap between observation windows. Hence, if any calculated time constant is longer than observed, one cannot argue that some experimental observation window was limited (as it was suggested in [23] for the case of ethylene).
• Similar as transient absorption or photoelectron spectroscopy, multiphoton ionization is vertical. When the wave packet runs down the potential surfaces and kinetic (vibrational) energy is released thereby, most of this energy is therefore taken up to the ionic surface(s) and causes an increase of fragmentation in the ion (with the mass peaks remaining sharp). This in turn provides a rough estimate of the energy of the corresponding location in the neutral. By contrast, in absorption or photoelectron spectroscopy the excess energy gives rise to hot bands or broadening and overlapping bands so that assignment can be difficult.
• As said, the mass peaks are sharp. No spectral deconvolution is necessary. This contributes to a good signal‐to‐noise ratio. We usually monitored the multiexponential decays over four orders of magnitude, sometimes more (in cyclohexene seven [24]; part of the range extension was done by increasing the pressure. This would not be possible in a molecular beam). This not only helped to decompose the decays into their components but also to isolate minor modulations, even in weak tails. Only in this way it was possible to detect slow oscillations with periods longer than a time constant. The correspondingly precise time constants and oscillation frequencies also allowed for a reliable determination of small isotope effects such as in ethylene (Section 4.1); in a previous attempt [25], the deuterium effect on the time constants of ethylene was below the detection limit. The deuterium isotope effect on oscillation frequencies and time constants was often very helpful in assignments and provided valuable information on the wave packet path. By contrast, diffraction experiments cannot detect involvement of hydrogen motion.
• Sometimes, we used longer wavelengths for probing instead of 800 nm. This helped to discriminate better between the different observation windows (which are energy windows). It also made the oscillations more prominent (examples for both in DMABN, Section 6.1).

## 3 Z/E Isomerization of Polyenes via a Hula‐Twist CoIn

### 3.1 Nonpolar Conjugated Polyenes

7‐Dehydrocholesterol on UV irradiation opens its B ring (type 1,3‐cyclohexadiene) in an electrocyclic reaction to form the *Z*‐triene *previtamin D* (Scheme 1). This in turn can undergo a photochemical *Z*/*E* isomerization to tachysterol. In order to analyze an ultrafast transient absorption experiment, using spectra also from an independent source, we studied these reactions also in a frozen matrix at 90 K [26]. We assumed that ring opening gives rise exclusively to the *cis*,*Z*,*cis* conformer of the triene. Surprisingly, this conformer isomerizes to the *trans*,*E*,*cis* conformer of tachysterol. (The conformers were identified by their UV spectra, assigned previously by their circular dichroism). The C_6_=C_7_ double bond and one single bond (C_5_—C_6_) both seem to rotate simultaneously by 180°. An alternative description is that only one CH group (C_6_) rotates out of plane, whereas the end groups are only reoriented in the plane. This type of motion was named “Hula‐twist isomerization” by Liu [27].

**SCHEME 1:**
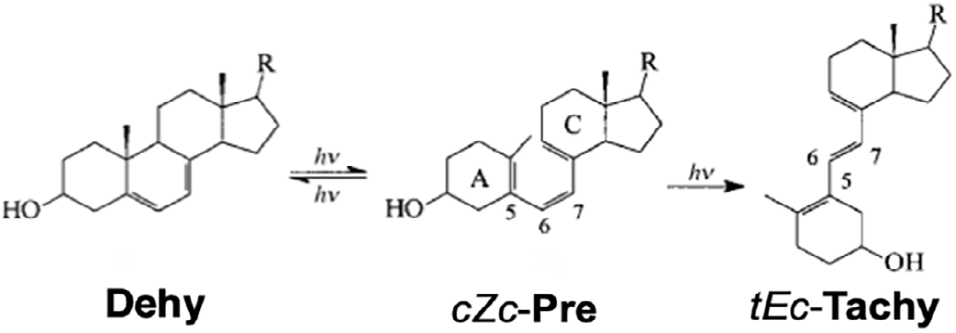
The *cis*,*Z*,*cis* conformer of previtamin D is prepared by photochemical ring opening of 7‐dehydrocholesterol (Dehy) in a cold matrix, where conformer isomerization is prevented. Its subsequent photoisomerization results primarily in the *trans*,*E*,*cis* conformer of tachysterol [26].

Before, it was believed that electronic excitation prevents rotation of single bonds in conjugated molecules. This would be consistent with the very successful principle of non‐equilibration of excited conformers [28, 29, 30]. However, as repeatedly stated (e.g., in [31]), (1) our reaction scheme is fully consistent with this principle, as one reactant conformer produces a product conformer (or conformers) different from that (those) produced from another reactant conformer, and (2) the products are formed not in the excited state but on passing from the excited to the ground state via a conical intersection.

Garavelli, Olivucci and coworkers calculated by CASSCF for *nonpolar conjugated polyenes* a 2A/1A CoIn (a MECI actually) with a “kink,” in which the angle at one carbon is reduced from 120° down to 90° (with less reduction for longer polyenes) [32, 33]. The angle reduction lifts this carbon (C_2_) out of plane, as if a partial bond would form between the two neighbor carbons (C_1_, C_3_). Instead of “kink CoIn,” also the name “Hula‐twist CoIn” seems justified [33]. Our observed (Hula‐twist) products thus support the calculated structure at the CoIn [26]. Liu and coworkers then found (in a frozen matrix, as in our study) many more Hula‐twist products in cases, where simple double‐bond flip would deliver distinguishable products (reviews[34, 35, 36, 37, 38]) (see also Section 3.2.1).

The C_1_–C_3_ attraction (C_5_–C_7_ in Scheme 1) is an example of the concept of driving forces. This force was interpreted in [39] by π interaction of the LUMO + 1 orbital in the 2A state. A singly occupied HOMO – 1 orbital assists it, too. In fact, the two‐electron excited 2A state contains around 25% HOMO → LUMO + 1 excitation and the same amount of HOMO – 1 → LUMO excitation. This explains also why highly polar double‐bond systems (such as the protonated Schiff base of retinal) do not follow a Hula‐twist path: Their lowest excited S_1_ state is ionic at all geometries, of HOMO → LUMO type, no LUMO + 1 involved, hence not of the type of the 2A state of nonpolar systems. It may also explain, why no Hula‐twist CoIn was found in a calculation on butadiene [40], which did not include the LUMO + 1 orbital in the active space.

The idea of such a driving force also allows to understand more details.

In nonpolar conjugated polyenes the quantum yield for *E*/*Z* isomerization becomes lower and lower, the longer the chain length, and is even not detectable, when the number of double bonds is of the order of 10. This was rationalized [41] by the C_1_–C_3_ attraction becoming weaker, if the LUMO + 1 orbital is more delocalized. As a consequence, the C_2_ atom is less lifted out of plane so that in the CoIn branching toward the reactant (toward internal conversion) is more favored over (Hula twist) isomerization. The smaller excursion of C_2_ was deduced in [41] also from considering the intersection of the 2A and 1A surfaces, when these two states approach each other at longer chain length.

As the excursion of C_2_ from the plane is (much) less than 90°, the CoIns for *cis* → *trans* and *trans* → *cis* do not coincide. This rationalizes why the quantum yields of forward and backward reactions sum up to less than 1, as said before [31].

This intersection (CoIn) lies usually (exception: butadiene) a little bit higher than the 2A minimum [41]. Hence, the wave packet requires an activation energy *E*_a_ to reach the CoIn, being highest for octatetraene. The mentioned consideration of intersecting surfaces suggested a linear relationship between *E*_a_ and the energy *E*_00_ of the 2A minimum. This results in lifetimes for internal conversion, which drop exponentially not only with *E*_a_ but also with *E*_00_. Compared with the previous interpretation of the *exponential gap rule*, this model removed several inconsistencies (such as an unphysical pre‐exponential factor) and explained further observations [41].

The Hula‐twist CoIn and the CoIn for the electrocyclic *butadiene *⟷ *cyclobutene* isomerization are geometrically similar (Section 4.3.2). This similarity played an important role in the explanation, why photochemical ring opening of cyclobutene derivatives sometimes goes to the anti‐Woodward–Hoffmann direction (Section 4.3.2).

In hemithioindos with substituents that block thermal rotamerization, it was possible to detect separately the products of Hula twist, of double‐ and of single‐bond flip and attempted isomerization (internal conversion) [42], see Section 3.2.

### 3.2 Objections and Rebuttals

#### 3.2.1 Is a Confined Volume Necessary for Hula Twist?

Liu originally suggested Hula‐twist isomerization for rhodopsin to explain how its chromophore (the protonated Schiff base of retinal) can cis‐trans isomerize within the confined volume of the opsin matrix [27]. (As said, this system does not follow this path, although there is a temporary twist of several bonds according to calculations [43, 44, 45, 46]). Also in the many examples of nonpolar polyenes, observed by them and others, they said, Hula twist will only occur, if the “normal” “lower‐energy” simple double‐bond flip will be prevented [34, 35, 36, 37, 38]. We said (e.g., in [31]), however:

1. How can a “higher‐energy process” proceed at the low temperatures of the matrix?
2. The quantum‐chemical calculations predict that Hula twist (via a CoIn) is the lowest‐energy path. (Defendants of one‐bond flip usually do not say, along which coordinate the molecule finds out from the perpendicular minimum down to the ground state. Note that vertical relaxation is not possible in ultrashort time, see Section 2.1).
3. Cyclic dienes such as 1,3‐cyclo‐octadiene and ‐heptadiene can only cis‐trans isomerize via simultaneous torsion of a double and a single bond; the lower‐energy path involves the central single bond (Section 5.4.2) and is hence Hula twist. They do so in the gas phase with the same ultrafast speed as, for example, the open‐chain hexatriene [47].
4. Also isolated molecules such as cis‐stilbene in the gas phase (Section 3.2.2) or hemithioindigos in solution (end of Section 3.2.2) can follow the Hula‐twist path.
5. Alternatives to Hula twist exist and are sometimes chosen by molecules (Section 3.2.3) but are no contradiction.

#### 3.2.2 Stilbene

We studied by ultrafast spectroscopy the cis‐trans isomerization of *cis‐*stilbene in the gas phase [48]. We found, it leaves from the perpendicular (2A) minimum about 6 times faster than a derivative ("stiff stilbene"), in which rotation of the single bonds is blocked. That means, Hula twist is the lower‐energy path. A further evidence from stilbene [48] deserves an additional comment:

Hochstrasser and coworkers investigated the same reaction by polarized spectroscopy in solution to find out the orientation (represented by the line connecting the benzene ring centers) of the product trans‐stilbene compared with the reactant cis‐stilbene [49, 50, 51]. They found a reorientation by 35° instead of the 60° that were expected for a simple double‐bond flip. They did not find an explanation. But 35° is just the reorientation, when the molecule has twisted its double bond by 90°. If then the molecule continues by rotating only one of the CH groups, the benzene–benzene axis remains fixed in space. We concluded [48] that stilbene (not a typical conjugated polyene) follows an asynchronous Hula‐twist path: first a 90° C=C twist and then with rotation of C=C and one C—C together toward the CoIn and further.

Hochstrasser and coworkers also determined the relative orientation of dihydrophenantrene, the product of conrotatory electrocyclic ring closure starting from cis‐stilbene [52]. They found a very similar angle of reorientation. This suggests that also in this reaction, there is first a 90° twist of the ethylenic double bond and that the two reactions branch in the perpendicular minimum; they would differ just in the direction of the subsequent rotation of the phenyl group. This would imply that ring closure would also occur from *trans*‐stilbene. Such a one‐photon reaction from trans to ring‐closed products has apparently not been observed, in contrast to molecules, in which the phenyl groups are replaced by naphthyl or the like (review: [53]). Such observations were traditionally interpreted by initial adiabatic trans* → cis* conversion, followed by ring closure [53]. But at the end, the review [53] points to high‐level calculations, which in fact show a path from the perpendicular minimum to the ring‐closed dihydrophenanthrene. (The viability may, however, depend on the detailed energetics).

A different answer to the problem may be found, if one considers involvement of additional coordinates. In fact, trajectory (ab initio multiple spawning) calculations of the Martínez group found that the bifurcation to the two channels happens much before (after 150 fs, i.e., before leaving the fluorescent region), on the other hand “following a rapid initial ethylenic torsion” [54]. (Also the calculation of Liu, Xia and Zhang [55] shows a strong C=C twist before branching toward the cis‐trans and ring‐closure channels). They also found two separate lifetimes, the shorter one (250 fs) for the ring closure and the longer one (300–500 fs) for cis‐trans isomerization, indicating two separate stationary states (S_1_ minima). Their excited‐state lifetime (520 fs) is fully consistent with our measurements (300 + 160 fs [48]). (Such calculations were also used to simulate time‐resolved photoelectron spectra [56], were the authors saw a signature of the perpendicular minimum. Its measured lifetime seems a little bit longer than ours (160 fs) but shorter than calculated. A calculation, primarily devoted to assign Raman spectra assigned to the perpendicular minimum [57] found an early branching to the CoIns for *cis*‐*trans* isomerization and ring closure).

The paper of the Matínez group [54] is full of very illustrative information on the movements of the different atoms. They find three CoIns: one with C=C twist, one‐sided pyramidalization and partial H migration, which funnels most trajectories down (to *cis* and *trans*) stilbene, a similar one with partial phenyl migration, collecting a few percent of the trajectories (same products) and one for the electrocyclic ring closure (7% of the trajectories). The one with the partial phenyl migration is a Hula‐twist type CoIn, which we claim to dominate. One of our two main arguments (above) was the orientation of products relative to the reactant. Probably this information is also hidden in their data; it would be helpful to check. The calculations are based on CASSCF with two electrons in π and π*, i.e., in the HOMO and LUMO. We argued (see above) that participation of LUMO + 1 brings about the attractive C_1_–C_3_ interaction, which facilitates to reach a Hula‐twist CoIn. Perhaps inclusion of this orbital would modify the theoretical predictions.

Already before, in a systematic computational search for stilbene MECIs, the Martínez group found cis and trans “kinked” CoIns (analogous to the kinked or Hula‐twist CoIns of polyenes, Section 3.1) besides two for hydrogen migration and one for the electrocyclic ring closure [58]. For the further path down from the kink CoIns, the authors say that the ortho positions (C_1_ and C_6_) of the two phenyl groups “land on the same side”; this is typical for Hula twist, in which only the central part of the molecule rotates. (Nevertheless, the authors associate these CoIns with one‐bond flip and a higher‐energy H‐migration CoIn with Hula twist).

In another earlier paper, Tomasello, Garavelli, and Orlandi investigated the double‐bond isomerization of stilbene by the RASSCF method [59]. In their calculations, the molecules prefer the twisted‐pyramidalized CoIn, although some CCH bending also takes part.

The Arai group found a photochemical trans‐cis‐isomerization of a stilbene, in which dendrimer‐type rests are attached to the phenyl groups. With such bulky substituents, the only possible path for trans‐cis‐isomerization is Hula twist [60]. They found nearly the same fluorescence lifetimes and isomerization yields as in unsubstituted trans‐stilbene. This indicates that the wave‐packet path is the same in both molecules and that Hula twist is at least competitive in stilbene.

S_1_/S_0_ CoIns of Hula‐twist type and pericyclic type were expected, if the two‐electron excited 2A state (S_1_ near the perpendicular minimum) is covalent (in valence‐bond notation), such as the 2A state in polyenes. If this state is zwitterionic (as in alkenes), the CoIn is usually expected to have—in addition to a 90° twist—one pyramidalized carbon with a negative charge, with sp^3^‐like angles; it would lead to flipping of just a double bond. The five‐membered ring of stiff stilbene (mentioned above) favors such angles; the barrier for leaving the S_1_ minimum will thereby be reduced, if S_1_ is zwitterionic. We observed the opposite. Hence, we concluded [48] that in the gas phase, the perpendicular minimum of stilbene seems to belong to a covalent two‐electron excited 2A state (and stilbene leaves this minimum on a Hula‐twist path via a minor barrier). On the other hand, the calculations by spin‐flip density‐functional theory of Minezawa and Gordon classify the stilbene 2A state as ionic [61]. That the perpendicular minimum has some zwitterionic character in polar solution is demonstrated by the fact that it can be trapped by addition of the solvent methanol (see, e.g., [62]).

In solution, the situation may differ from the gas phase: An ionic (1B or 2A) state can be lowered by a polarizable (1B) or polar (1B, 2A) environment. In fact, Kovalenko and coworkers investigated the perpendicular minimum of several substituted stilbenes in different solvents (e.g., [63, 64]); some of their results might be interpreted by such polarity effects.

A feature of the Hula‐twist CoIn postulated for stilbene [48] deserves interest: The reaction is only half finished at the CoIn (as always); as suggested, the wave packet can continue in this direction and thus finalize the Hula twist (as observed by the polarized spectroscopy), or one of the two twists (or both) may be prevented from completion by external or internal effects (the latter by the effect of accumulated momentum). Thus, from one CoIn point, not only Hula twist is accessible but also double‐bond flip (“attempted Hula twist”), single‐bond flip (rotamerization) and internal conversion [48].

Such a type of CoIn was also suggested for the *hemithioindigos* [65]. All four reaction types—double‐bond flip, single‐bond flip, Hula twist, and attempted isomerization (internal conversion)—were experimentally detected [42]. Observation was facilitated by the substituents, which allowed the authors to identify the atrop‐isomers separately. Also charge‐transfer transitions are possible in these systems [42, 65]. On the other hand, ultrafast transient absorption of hemithioindigos was interpreted in terms of three excited states, whose relative energies was influenced by substituents (some of them inducing pre‐twist) and by solvents; from these states, different CoIns seem to be accessible [66].

#### 3.2.3 Alternatives to Hula Twist and Previtamin D Isomerization Revisited

The group of Saltiel presented some cases of simple double‐bond flip and a bicycle‐pedal isomerization in short‐chain polyenes and derivatives (e.g., [67, 68, 69]). (But note that the double‐bond flip is only assumed, e.g., in [68]. The corresponding products could equally well result from Hula twist, which would only lead to a different conformer: rapid rotamerization in solution wipes out the difference by conformer equilibration). It is conceivable that the molecule can cross the intersection space at minima (or locations) different from the Hula‐twist MECI—or the same CoIn allows also double‐bond flip via an attempted Hula twist, as explained in Section 3.2.1. Anyway, after a 90° double‐bond twist, an additional distortion is needed to leave the minimum toward a CoIn, be it attempted Hula twist or something similar as in ethylene or alkenes. (Note also that for the bicycle‐pedal mechanism a calculation finds for a similar compound in solid state only an excited‐state (adiabatic) path [70]). The alternatives will have the best chance to compete, where Hula‐twist needs the highest activation energy; this is octatetraene (S_1_ lifetime >10 ns) among the unsubstituted polyenes [41]. It may also play a role that for the shortest chain lengths (dienes, trienes) the covalent 2A and ionic 1B potentials are very close to each other. The latter is often assumed to favor pyramidalization in the CoIn and double‐bond flip, the former Hula twist.

The Saltiel group also repeated our previtamin D isomerization in a cold matrix [26], using principal‐component analysis of the UV spectra and in part also fluorescence to identify the conformers [71, 72]. They were able to detect also side products. The main conformers of the E isomer tachysterol were the same as in our experiment. However, in the ring opening of dehydrocholesterol or lumisterol to prepare the *Z* isomer previtamin D near liquid‐nitrogen temperature, they believed to see three conformers (mainly *trans*,*Z*,*cis*, no *trans*,*Z*,*trans*) instead of the *cis*,*Z*,*cis* conformer expected (and assumed by us). This would mean that in the ring opening, one of the two single bonds would rotate by 180°, perhaps in the hot ground state of the product due to recoil. The following *Z*/*E* isomerization would then lead to the three observed tachysterol conformers by a double‐bond flip, not a Hula twist, as the authors stated. However, a rebuttal pointed to a poor signal‐to‐noise ratio for the previtamin D spectra, so the identification of the primary conformer is not certain [31]; more importantly it pointed to a time‐resolved circular‐dichroism experiment [73] (Figure 2), which proved that on ring opening there is no rotation of a single bond, so that the product is in fact the *cis*,*Z*,*cis* conformer of previtamin D and the ensuing photoisomerization follows a Hula‐twist path. The side products were explained on the same basis [31], and many additional observations and arguments were presented that are more consistent with Hula twist than with double‐bond flip.

**FIGURE 2:**
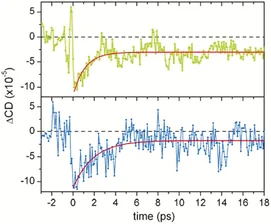
Time‐resolved circular dichroism (CD) upon photochemical ring opening of 7‐dehydrocholesterol to previtamin D, measured in [73] with 300 and 285 nm (upper and lower trace, respectively) for photolysis and probing. The shown ΔCD stays negative over at least 18 ps, which proves that photolysis does not form substantial quantities of conformers different from the expected *cis*,*Z*,*cis*‐previtamin D. The initial decay is believed to reflect the product formation time (ca. 2 ps) from S_1_ of the reactant. Details are discussed in [31]. Reproduced from [73] with kind permission of ACS.

To summarize the cis‐trans isomerization of polyenes: A strong experimental support of the calculated Hula‐twist type CoIn are the (conformers of the) end products. This type of CoIn provides also paths to flipping of the C=C bond alone (attempted Hula twist), to rotamerization and to internal conversion. The calculations were interpreted in terms of an attractive 1–3 interaction, providing a *driving force* for leaving the 2A minimum. This force involves the LUMO + 1 orbital, which contributes to the 2A state. The C_1_–C_3_ attraction also decreases the CCC angle at the enclosed C_2_, which is therefore temporarily rehybridized (pyramidalized). Stilbene seems to be a limiting case between alkenes and polyenes: It can photoisomerize on an asynchronous Hula‐t‐twist path but also exhibits (in small quantum yields [62]) carbene reactions and addition of alcohols.

## 4 Ethylene and Alkenes

### 4.1 Ethylene

Ethylene has a strong absorption band in the vacuum UV with maximum near 162 nm (7.65 eV) and a long‐wavelength wing extending to beyond 200 nm (6.2 eV). It is dominated by a π → π* transition, superimposed by a Rydberg (π → 3s) transition, which is more structured. The ππ* state is π antibonding. Its energy is therefore lowered by CC stretching and twisting, by about 2 eV at 90° twist. Also rehybridization (pyramidalization) reduces the overlap of antibonding orbitals; this is the only possibility (besides CC stretching) for some cyclic olefins such as norbornene (bicyclo‐2.2.1‐heptene), whose double bond cannot twist.

Excitation of the ππ* state causes cis‐trans isomerization (in ethylene detectable, if it is isotopically labeled) and typical products of singlet carbenes that result from hydrogen or—in alkenes—alkyl migration (reviews [74, 75, 76, 77]). Hence, the intersection space must present easily accessible regions, which not only are half‐way between *cis* and *trans* (that is, with a twist near 90°) but are also midway along the migration path, perhaps with an H or alkyl group forming a bridge over the previous CC double bond (with a preceding C=C twist).

The existence of an easily accessible CoIn is proven by the measured ultrashort excited‐state lifetimes (Figure 3). In our lab, we found that ethylene leaves from its (perpendicular, *D*_2d_) S_1_ minimum within 17 fs [7] (consistent with earlier experiments with less time resolution [25, 78, 79, 80]), cyclohexene within 43 fs [24], and norbornene within 52 fs [81]. That is, from S_1_, the molecules pass through an S_1_/S_0_ CoIn.

**FIGURE 3:**
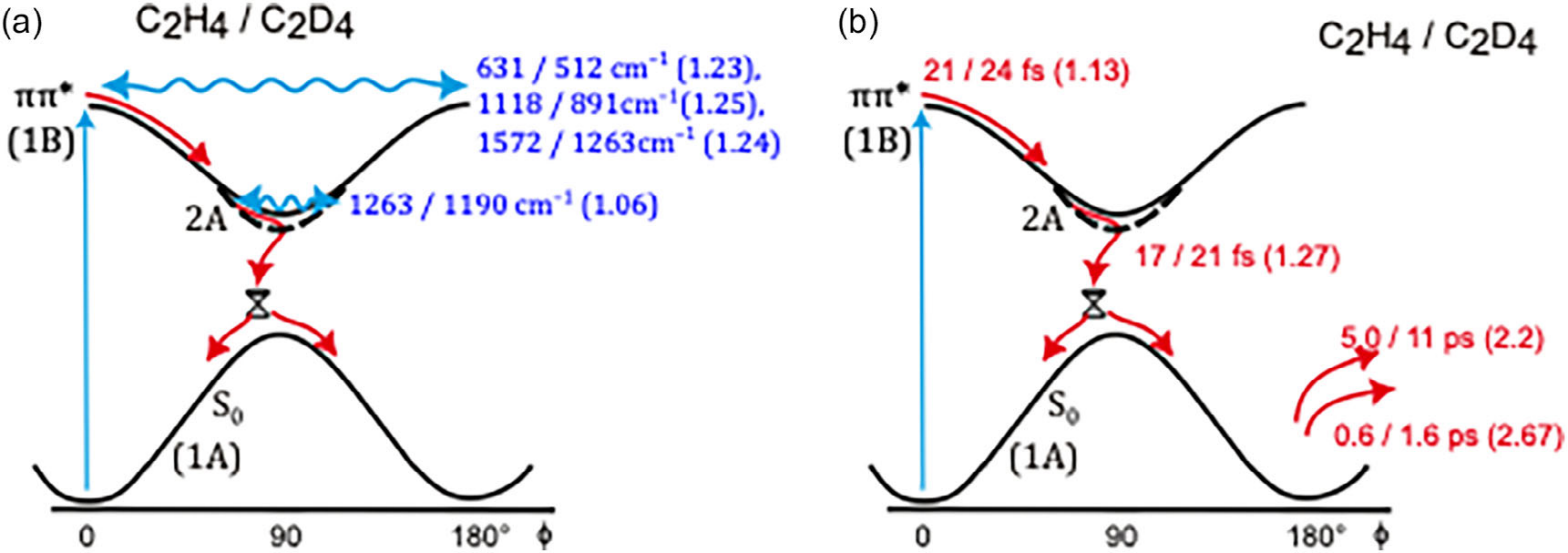
Isotope effects (ratios in parentheses) on the vibrational wavenumber of coherent oscillations (a) and the time constants (b) in ethylene; in the Franck–Condon regions, the oscillation wavenumbers belong to the hindered free rotation (“twist”), the CC stretch and a combination vibration, near the perpendicular minimum to the CC stretch. The two‐electron excited state (2A) is shown as a broken line. The figure shows only one coordinate, the C=C torsion. According to the text, the direction to the 1B/2A CoIn leads out of the drawing plane, initially toward H migration, followed by one‐sided pyramidalization. Accordingly, there are two products (from where the two picosecond processes start): ethylene (with 180° or 0° torsion) and ethylidene (the result of H migration). Note also that the traveling times *τ*_1_ and *τ*_2_ are shorter than a vibrational period of twist and CC stretch vibration. The figure is based on [7], modified by indicating the 2A state, with kind permission of ACS.

As in most of our time‐resolved experiments, probing was done also in ethylene by multiphoton ionization, measuring the transient yields of molecular and fragment ions (time‐resolved mass spectroscopy, Section 2.2). Monitoring the yields over three to four orders of magnitude, we could follow the path over different observation windows L_*i*_ from the Franck–Condon region (L_1_, 7.6 eV in ethylene, lifetime 21 fs, preceding the 17 fs for leaving L_2_, the S_1_ perpendicular minimum, Figure 3) down to the ground state. The L_*i*_ differ by their energy of ionization and were distinguished by the degree of fragmentation and sometimes by the order of multiphoton ionization. The latter was also used to identify two slower time constants in L_3_, (600 fs and 4.5 ps) as two ground‐state processes, which must be assigned to the two known photoproducts, hot ethylidene and hot ethylene. The time resolution (11 fs) and signal‐to‐noise ratio were good enough to detect coherent oscillations (which confirmed expected slopes toward CC stretching and twisting) and the partly very small deuterium isotope effect (Figure 3). For the initial motion (L_1_) the unexpectedly small D effect was ascribed to intimate coupling of CC stretch and twist (as in a parametric oscillator); in fact, the isotope effect in the observed coherent oscillations was also unusually small in the twist vibration and unusually large in the CC stretch vibration. The combined motion along CC stretch and twist was also found in the quantum‐dynamics calculations of Viel and coworkers [82]. The energetic dominance of acceleration toward CC stretch was again underlined in [83]; perhaps it is underestimated in the calculations, which so far predict too long lifetimes. Because of our better signal‐to‐noise ratio and time resolution, our values for the isotope effects are probably more reliable than the larger ones (near 1.5) reported recently by Boyer and coworkers [84].

Special interest deserves the leaving from the perpendicular minimum to the CoIn: The time for it (*τ*_2_) is 21 fs for C_2_D_4_, a factor of 1.27 longer than the 16.5 fs for C_2_H_4_. If the direction of motion would be only pyramidalization, the square root of reduced masses would predict a ratio slightly below 1.4. Obviously, a CC stretch motion (expansion or compression) must substantially contribute. Interestingly, in this region (L_2_) the frequency of the CC stretch oscillation (1263 cm^–1^, 1.09 times larger than in C_2_D_4_, an isotope effect just like in the pure CC stretch vibration in S_0_) has increased by 13% (force constant by 26%) compared with L_1_. A strengthening of the CC bond is expected, if it is bridged by a hydrogen but not expected with pyramidalization alone. That is, the wave packet moves from the perpendicular minimum toward the CoIn (toward the IS) along a coordinate that comprises an H bridge formation with accompanying CC bond strengthening. But pyramidalization also takes place, as shown by the time‐resolved X‐ray spectroscopy of Neville and coworkers [6] (mentioned in the Introduction and to be discussed below).

The evidence from our experiment would be less conclusive, if our observation window were localized in the region of the perpendicular minimum (where the CC bond strength has already recovered to some extent compared with the Franck–Condon region, where π repulsion is stronger). But what we observe are energy windows, and the energy drops strongly only on passing the CoIn. The window L_3_ (time constants *τ*_3_ and *τ*_4_), immediately following L_2_ (with the lifetime *τ*_2_ = 17 fs), clearly belongs to the ground state, as to conclude from the increased number of photons required for ionization and from the increased ionic fragmentation. Between L_2_ and L_3_ there cannot be any gap that would elude detection by ionization (as already pointed out in Section 2.2). Hence, L_2_ clearly extends from the perpendicular minimum to the CoIn.

For the path from the perpendicular minimum to the CoIn, there seems to be a conflict between our measured isotope effects, supporting a hydrogen bridge without pyramidalization, and the evidence for one‐sided rehybridization found by X‐ray spectroscopy [6]. The conflict can be solved by the assumption that an initial formation of an H bridge is followed by a faster pyramidalization. In kinetics, a much faster process after a slower one can usually not be seen separately if monitored by a single signal.

H migration is further discussed in the context of the wavelength dependence of alkene photochemistry (Section 4.3).

It is interesting that the quantum‐dynamics calculations of Viel and coworkers [82, 85], that used only six dimensions, also found that from the perpendicular minimum the initial path is toward an H bridge, not toward pyramidalization. As a reason they see the nonzero slope toward scissoring (in other words, scissoring is a component of the NADC vector of the 1B/2A CoIn), in contrast to the initial zero slope toward rehybridization. This calculation also predicted recurrences that coincide very well with the coherent oscillations observed by us [7]. Also the high‐level calculation of the Lischka group [86] predicts that ethylene leaves from the perpendicular minimum initially forming an H bridge; this is followed by a rapid pyramidalization, maintaining the bridge. (See also Section 4.2).

### 4.2 Other Experiments and Calculations

Previous measurements of the time constants with less time resolution [25, 78, 79, 80] were consistent with ours (see [7]). Also, later experiments confirmed some of them, again within the limits of their time resolution and/or signal to noise ratio. Thus, by transient one‐photon ionization measuring ion yields for probing, Peralta Conde and coworkers found 24 and 21 fs for the first two time constants [87], and Tao and coworkers 21 fs for the first one [23]. In two experiments with time‐resolved photoelectron spectroscopy, slightly longer lifetimes were found for the Franck‐Condon region [88, 89], presumably due to a slightly broader observation window in this method. Kobayashi and coworkers also observed a weak recurrence at 18 fs, corresponding to a coherent oscillation with wave number (18 fs × *c*)^–1^ = 1850 cm^–1^, slightly higher than our fastest oscillation (1572 cm^–1^), which we assigned to a combination vibration of CC stretch (1118 cm^–1^) and twist (631 cm^–1^). They assigned it to the twist. Our slower oscillations were observable only on including the weak temporal tails. We assigned the lowest wavenumber, 631 cm^–1^, to the twist, actually a hindered free rotation in this energy region; this is in full agreement with spectroscopic knowledge (see the discussion in [7]).

In a more recent remarkable extension of their previous work [88], the Suzuki group used a high harmonic with 21.7 eV photon energy to generate the photoelectron spectra (resolution 32 fs) [90]. In this way, they were able to monitor the process down to the ground state; the photoelectron energies gave some indication of the energies of the probed states. They report two consecutive excited‐state processes with time constants 23 and 25 fs, similar to ours (21 and 17 fs). They found also two ground‐state time constants (0.87 and >5 ps) and assigned both to hot ethylene and consecutive products (the expected photoelectron spectrum of ethylidene was not separable from that of hot ethylene), whereas we attributed our shorter ground‐state time (0.6 ps) to ethylidene, whose hydrogen back migration has a minor barrier at most. Our assignment was based on a kinetic argument: Hot ethylene can show only one time constant, that of the rate‐determining first step; faster consecutive reactions would not show up. So, the second observed time constant must belong to the second known product, ethylidene. The longer time constant must belong to the ethylene decomposition, which is endothermic. A lifetime of 0.5–1 ps for ethylidene seems still long enough to be compatible with the observed product formation in bimolecular reactions in liquid ethylene. Our assignment is also supported by the observed fragmentation after ionization [7].

In a follow‐up work the Suzuki group also investigated C_2_D_4_ [84]. Compared with their (reevaluated and slightly changed) C_2_H_4_ data, they found for the excited‐state lifetimes 1.5 and 1.4 times larger values (as expected for pure hydrogen movements); but surprisingly for the two ground state processes, they found no isotope effect. This is in contrast with the lengthening that we found [7]: by the (small) factors 1.13 and 1.27 for *τ*_1_ and *τ*_2_, and for the two ground‐state processes more than a factor of 2, consistent with activated or even endothermic processes involving hydrogen. A difficulty of comparison is the difference in evaluation: The Suzuki group assumed a latency period (*τ*_lat_, no isotopic dependence) before the kinetic evolution. (In the sum *τ*_lat _+ *τ*_*i*_, the isotope effect would be reduced). Our analysis did not assume any latency time and used pure kinetic analysis in consecutive processes (resulting in sums of exponentials), modified by oscillations. Similarly, it is difficult to compare the results of Stert and coworkers [25]: They used Bloch equations for their analysis and, with pumping at 198 nm, found for *τ*_1_ and *τ*_2_ the values 10 and 15 fs for C_2_H_4_, and 10 and 20 fs for C_2_D_4_; but in view of their laser pulses of 130 fs duration, they consider the times to be equal within the error limits.

It seems likely that among the published isotope effects ours are more reliable, in view of our better time resolution and much better signal‐to‐noise ratio, as already said. Furthermore, the D effects on the observed oscillations were also consistent with each other, e.g., the surprisingly small effect on the twist and seemingly too large effect on CC stretch in the Franck–Condon region, because the two vibrations are mixed. This mixing of motion direction in turn is consistent with the very small change of *τ*_1_ on replacing H by D.

Quantum‐chemical calculations of the dynamics still have difficulties to reproduce the short times [86, 91]. The problem is reduced by inclusion of dynamic electron correlation, to some extent also by including CH repulsive states [86, 92]. It would be interesting to see their prediction for the various isotope effects, also as a test for the relative importance of acceleration toward CC stretch and twist.

From the observation [74, 75, 76, 77] that both cis‐trans products and carbenes are photochemical products (i.e., not the results of hot ground‐state reactions, which are negligible in condensed phase), it must be concluded that the S_1_/S_0_ CoIn or IS is displaced and/or provides slopes in both directions. Hence again, already the observed products pointed to the nature of the CoIn and slopes, although the time‐resolved spectroscopy yielded much more information.

In fact, already early quantum chemical calculations (summarized, e.g., in [85, 93, 94]) found a CoIn with an H bridge over the two C atoms (with preceding C=C twist), that is, with hydrogen migration half accomplished. This was confirmed also by later calculations [86, 92]. The further path leads to both, cis‐trans isomerization and ethylidene (and back to the reactant). Another important coordinate resulting from the calculations was one‐sided pyramidalization. This deformation on the way from leaving the S_1_ minimum was confirmed by transient X‐ray spectroscopy [6], as already mentioned. Resonance Raman spectroscopy found displacement not only toward CC stretch and twist but also toward pyramidalizaton and CH_2_ scissors coordinates [95]. Viel and coworkers also pointed to the importance of the one‐sided scissors deformation in their theoretical investigations [85]: It is the nonadiabatic coupling vector, causing a splitting of the ππ* and π*^2^ states near the *D*_2d_ geometry already in first order. In their high‐level (MR‐CISD) calculations including also some Rydberg and σ* orbitals, Barbatti and coworkers [94] explored a larger part of the IS. They found that from the twisted minimum of S_1_, the region of the H‐bridge CoIn can be easily reached on a flat down‐slope. But from there, pyramidalization (with maintaining the H bridge according to their Figure 10) further lowers the energy. In later publications [86] they present a “hydrogen migration CoIn” with H bridge and 90° twist but no pyramidalization and a “twisted pyramidalized CoIn” without H bridge, besides an ethylidene CoIn (Figure 4). (A validation of the calculations on ethylene potentials and CoIns can be found in the review of Szalay and coworkers [96]). It should be noted, however, that each of the two CoIn structures can lead in the calculations to both, H migration and cis‐trans isomerization; hence, the two reactions do not necessarily indicate that the path leads through two different CoIn structures. It is also worth noting that in some calculations, the migrating H comes from the pyramidalized group; in others, this group receives an H from the other side. The wavelength dependence of alkene photochemistry (increased carbene formation from Rydberg states) suggests that pyramidalization substantially enhances hydrogen migration according to Section 4.3.

**FIGURE 4:**
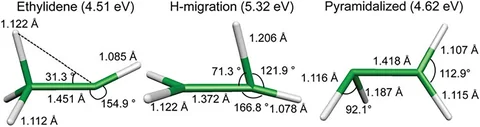
S_1_/S_0_ MECI structures of ethylene, calculated at the MRCI level [86]. In the text, it is predicted that ethylene leaves from the perpendicular minimum via a partial H migration (middle figure), after which follows a rapid pyramidalization, maintaining the H bridge. Reproduced from [86] with kind permission of Taylor and Francis.

In fact, a more recent computational search finds a twisted‐pyramidalized geometry with an unsymmetric H bridge (with the pyramidalized group as the H donor) as the IS minimum (slightly lower than pyramidalized without H bridge), the twisted H bridge without pyramidalization at higher energy [97]. Another systematic search considers “twisted pyramidalized” and “H‐bridging” CoIns in ethylene as identical [58].

On the other hand, some quantum chemical calculations of the dynamics did not find an H‐bridge CoIn, pointed to the importance of the twisted‐pyramidalized CoIn and said that H migration is completed in the excited state, where it leads directly to a degenerate state (CoIn) of CH_3_CH, not far from its ground‐state geometry [91, 98]. (This CoIn results from Jahn–Teller splitting of the degenerate ethylidene ground state). They did not notice an earlier S_1_/S_0_ degeneracy during the migration path, in contrast to other calculations; the excited‐state population is expected to drop down very efficiently to S_0_ through such an earlier (H‐bridge) CoIn.

The latter work [98] presents quantum chemical simulations accompanying a pump‐probe experiment on ethylene (resolution 44 fs), where the pump was at 161 nm and the probe was in the XUV, and yields of the ions CH_2_^+^, CH_3_^+^, H_2_^+^ and H^+^ (besides the parent ion C_2_H_4_^+^ and others) were presented. The authors assign the early parts of the CH_2_^+^ and CH_3_^+^ signals to excited ethylene and the late parts (at times >100 fs, after the S_1_ has decayed) to the ground state. Surprisingly, then they suggest that the signals originate from two CoIn geometries: the twisted‐pyramidalized one giving rise to CH_2_^+^ and the ethylidene CoIn to CH_3_^+^; if so, the signals should last much shorter than 17 fs, the time we measured for traveling from the twisted S_1_ minimum through to the CoIn (or IS). It seems very suggestive, however, that the tail of the measured CH_3_^+^ signal (not present in their simulation) reflects the lifetime of CH_3_CH (which decays primarily by H back‐migration, also by H_2_ elimination). The rise time of the accumulated H_2_ (giving the H_2_^+^ signal), around 200 fs (184 fs is given in [99]), should be the same, and seems to be so. Hence, another time‐resolved experiment supports CH_3_CH as a photoproduct. (It is, however, not clear, why they find a lifetime shorter than our 600 fs, and no deuterium isotope effect, in contrast to us. Perhaps one must wait for better signal‐to‐noise ratio).

The simulations seem to me so far not to be suitable to reproduce the signals in this work: They show too slow rise time (a known problem, see above or [100]), a second maximum not shown in the measurements, too fast decay to 0; the calculation even show a fast decay to 0 for CH_2_^+^, whose late part was assigned to the ground state of hot ethylene. The very non‐exponential behavior of the calculated signals is also in contrast to our measurements, which could be analyzed as a sum of exponentials (sometimes with oscillatory components) over several orders of magnitude (also in [7]). It was already previously noted [83] that the calculations [91] predict ejection of an H atom with 27 km/s, corresponding to a kinetic energy of 3.7 eV, as if accelerated down from a CoIn; the energy determined by photofragment spectroscopy is 0.61–0.66 eV [101, 102, 103], as often observed for reactions in the hot ground state.

One can summarize, what to conclude from Sections 4.1 and 4.2 on the potentials and the S_1_/S_0_ CoIn or IS of ethylene mainly from experiments: The observed products require that the intersection region provides slopes (or/and displacements of preferred IS regions) in direction of both, cis‐trans isomerization, and carbene formation (hydrogen migration). The motion from the perpendicular S_1_ minimum to the CoIn region is suggested to involve an early formation of an H bridge, initially without (much) pyramidalization. The latter would follow and take shorter time, so that it is not kinetically resolved in our experiment. One‐sided pyramidalization is supported by X‐ray spectroscopy [6]. The H bridge is better compatible with the increased CC stretch force constant (oscillation frequency) and the small deuterium effect for the traveling time toward the last CoIn [7]; the D effect would be too small to be compatible with a dominance of pyramidalization. According to calculations [86, 92, 94], the path with an H bridge alone is energetically less favorable than with dominating pyramidalization. But it could be taken anyway, because on going around the first (ππ*/π*^2^ or 1B/2A) CoIn the wave packet is accelerated toward the NADC vector, a one‐sided scissors coordinate. If so, the wave packet does not follow the lowest‐energy path but is influenced by momentum effects. The acceleration at the first CoIn also rationalizes the short time of leaving from the perpendicular minimum, although the path from there is very flat.

The concept of driving forces was used at three locations: (1) In the Franck–Condon region the slope toward a twist is zero, which is in stark contrast to the ultrashort leaving time from there. But the down‐slope toward CC stretching is steep, and the intimate coupling of the two motions (inferred from the D isotope effects) also explains the rapid appearance of the twist. (2) An acceleration toward a hydrogen shift can be expected on crossing over from the 1B (ππ*) to the 2A (π*^2^) state near the perpendicular minimum. (3) After arriving in the 2A state, the molecule (with its 90° twist and beginning H migration) can further lower its energy by one‐sided pyramidalization. This stabilizes the molecule in this state (and destabilizes the ground state, so that the CoIn is approached). This is because in ethylene (and alkenes) the 2A state is zwitterionic [1], and the negatively charged carbon rehybridizes to sp^3^. In dienes and polyenes, the 2A state is not zwitterionic but covalent; if a pyramidalization is found there too, there must be another reason (e.g., Hula twist, as discussed with cyclooctadiene in Section 5.4.2).

### 4.3 The Wavelength Dependence in Alkene Photochemistry: A Momentum Effect

In alkenes, the photoproducts corresponding to those of ethylene are cis‐trans isomers and carbenes, the latter formed by hydrogen or alkyl 1,2‐migration. In addition, one observes 1,3‐sigmatropic shifts of an H atom or an alkyl group (reviews [74, 75, 76, 77]). The ratio of product yields depends on wavelength. Carbene products dominate on excitation of the Rydberg (π3s) state, whereas cis‐trans isomerization is preferred from the ππ* state, and a supposed higher third state was invoked that would favor 1,3‐sigmatropic shifts. The carbene formation from the π3s state was interpreted by the positive charge, left in the valence shell on Rydberg excitation, and the fact that 1,2‐migration is common in positive ions [74, 75, 76, 77]. However, before forming a product, the molecule passes over to the ππ* state, as revealed by ultrafast spectroscopy (see below). This finding seems to withdraw the basis for the dependence on the initial state.

Already in ethylene the Rydberg(π3s) transition slightly precedes (the Franck–Condon maximum of) the ππ* band. By alkylation, both states are lowered, but π3s more than ππ*. For example, in tetramethyl ethylene (2,3‐dimethyl‐2‐butene) the Rydberg absorption precedes the ππ* band, with negligible overlap, so that separate excitation is possible.

Because the ππ* state is π‐antibonding, its potential is lowered on twisting, whereas that of the π3s state rises slightly in this direction. Hence, the two surfaces intersect (conically), and population can flow from the Rydberg state with very little activation energy around the lower cone of the CoIn to the ππ* state. The activation energy rises a little, if the two states are more separate in the substituted molecules. This was explained before, for example in [104]. This group detected (and assigned) this path in methylated ethylenes by time‐resolved photoelectron spectroscopy and determined the lifetime of the π3s state and (after departure from it) that of the ππ* state. The lifetime in the ethylene Rydberg state—80 fs—was determined in our lab [7].

Hence again: If the population of the Rydberg state flows efficiently to the ππ* surface, how can the two states then differ in their photoproducts? We suggested in [17, 24, 81] that the wave packet keeps a memory over some time (or some path length on the potential), where it came from. This would work in the following way: As the two states have different symmetry (B_3u_ and B_1u_ in *D*_2h_ (ethylene), B_1_ and B_2_ in *C*_2v_ (simple alkenes)), for passing from one to the other state the molecule must be distorted in the direction of the nonadiabatic coupling (NADC) vector (symmetry b_2g _= B_3u_ × B_1u_ with ethylene or a_2 _= B_1 _× B_2_ in alkenes), as shown in Figures 5 and 6. This is the deflection of the wave packet on going around the CoIn, and the direction of an additional momentum imparted to it. For conservation of this momentum till the branching region (the last CoIn or IS), the potential must not further deflect the wave packet and must be sufficiently independent of direction (Section 2.1). In fact, the entrance region to the CoIn (or of the lower parts of the IS) of ethylene is rather flat in the calculations [94]. Hence, theory and experiment support each other in this respect.

**FIGURE 5:**
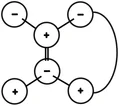
Distortion (b_2g_ in D_2h_ as in ethylene, a_2_ in C_2v_ as in cyclic alkenes) on going around the Rydberg(π3s)/ππ* CoIn, showing pyramidalization of both double‐bonded C atoms. From [17] (modified), with kind permission of Elsevier.

**FIGURE 6:**
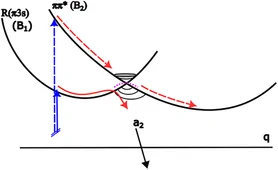
From the ππ* state, alkenes (symmetry *C*_2v_) initially follow a path (broken line) along a coordinate *q* that contains C=C stretch and twist as important components. (From the minimum, the direction changes, to find a CoIn with S_0_). The path from the Rydberg (π3s) state (solid line) is deflected, with acceleration to an a_2_ direction, which is a two‐sided pyramidalization according to Figure 5. The deflection prevents that the two paths merge, although they propagate on the same (ππ*) surface. The dotted line would correspond to wavelength‐independence but would be a symmetry‐forbidden path. “CC” indicates C=C stretch, “a_2_” can be C=C torsion or two‐sided pyramidalization as in Figure 5.

However, it would be desirable to see a more direct theoretical investigation of the wavelength dependence. One reason for this need is the fact that the b_2g_ coordinate in ethylene points to (two‐sided) pyramidalization, and this is not the direction, which on first sight is associated with hydrogen migration (although allowing it). A more favorable coordinate seems to be a scissors‐like motion of a CH_2_ group (after twisting and perhaps other deformations); two‐sided scissoring of symmetry b_1u_ would be the NADC vector for the CoIn between the ππ* (1B) and the π*^2^ (2A) states near the perpendicular minimum (Section 4.2). One may speculate about a cooperation of the two deflections: Pyramidalization on passing by the Rydberg(π3s)/ππ* CoIn will lower the (higher‐lying, zwitterionic) 2A state so that the 1B/2A IS is met earlier, at higher energy, and slopes will be steeper, including that toward scissoring. But a calculation would be better. Instead of this speculation, we previously suggested (e.g., in [17]) that it is the pyramidalized CoIn that leads to more carbene formation. In fact, also one calculation [58] considers “twisted pyramidalized” and “H‐bridging” CoIns in ethylene as identical, as mentioned.

It seems reasonable to postulate a momentum toward hydrogen (or alkyl) migration to explain the wavelength dependence: The two CoIns, twisted‐pyramidalized and twisted‐H‐bridge, do not differ in the twist angle (90°). Hence, a different push direction must be responsible for favoring one of the CoIns or directions.

A clear example of this mechanism is also the ring opening of cyclobutenes (Section 4.3.2).

To come back to the concept of driving forces: (1) Without the deflection at the CoIn, the two paths would merge, and there would be no wavelength dependence. That is, the force (slope) toward the NADC vector is needed, no matter what exactly follows. (2) It is said that that in alkenes (monoolefins), there is no force changing the direction of a wave packet arriving at the last CoIn, from where both cis‐trans isomerization and 1,2‐nigration (carbene formation) can take place; that is, a preceding momentum direction is conserved.

The suggested mechanism for the wavelength dependence would not work, if the molecule would thermalize in S_1_, because the information would be lost, where the wave packet came from. Hence, this kind of wavelength dependence requires some degree of coherence. This seems reasonable in view of the short measured times and the coherent oscillations observed in some cases.

#### 4.3.1 Calculations and Ultrafast Spectroscopy on Cyclohexene

On the basis of their CASSCF calculations for cyclohexene, Wilsey and Houk also point out that carbene formation and cis‐trans isomerization take place from the same potential surface [105]. (The calculations comprise the ππ* potential besides S_0_, not the π*^2^ state). This was the case even for the third type of reaction, the 1,3‐sigmatropic shifts. Interestingly, for the latter path, they calculated a small activation energy. This may explain why shorter wavelengths (higher photon energies) increase the yields of the 1,3‐shifts. On the other hand, considering the time constants near 50 fs found by us for cyclohexene [24] and norbornene [81], the activation energies must be really minor, at least in these two examples.

Calculations so far did not address the question, how a wavelength dependence can come about, although all reactions pass via the same lower excited‐state surfaces. The role of two‐electron excited states in olefin photochemistry has also not been clarified by them. Several calculations also find CoIns leading to dissociation or bond breaking, whereas such reactions are observed to occur only in the hot ground state [24] (Section 4.2).

The Stolow group investigated by time‐resolved photoelectron spectroscopy cyclohexene, comparing the results with 1,4‐cyclohexadiene and with CASSCF calculations [106]. The calculated MECIs were consistent with those of Wilsey and Houk [105]. Their spectroscopic method provided an estimate (independent of ours) for the energies of the observation windows. They found three on the excited potentials, one of them clearly lower than the first two (just as we found, too), followed by several windows of the hot ground state (we found one only, without a closer investigation). The regions of clearly different energy are probably separate minima (“collection wells,” each with a CoIn as exit) on the same excited‐state (S_1_) surface (consistent with the calculations [105, 106]). We originally suggested [24] two separate states, ππ* and π*^2^, where the latter would lie lower; but the higher minimum (responsible for the 1,3‐sigmatropic H‐shift) can also be part of the S_1_ surface, and it is conceivable that the ππ* and π*^2^ states are practically isoenergetic around this location. It should also be noted that calculations (such as CASSCF) without dynamic electron correlation usually places the two‐electron excited states too high; furthermore, the widely accepted model of van der Lugt and Oosterhoff (in the meantime augmented by CoIns [1]) uses such a state as collection well in pericyclic reactions, also in the case of 1,3‐sigmantropic shifts [107]. The calculations [105, 106] left open, where is the branching to the different CoIns leading to the various reactions. We associated the window with time constant *τ*_2_ to the path leading to 1,3‐sigmatropic hydrogen shift on the basis of the deuterium isotope effect.

Cyclohexene was also investigated by time‐resolved photoelectron imaging spectroscopy [108]. The authors were able to assign a signal and its anisotropy to the primary product cyclopentyl carbene (lifetime 400 fs in the hot ground state, in good agreement with our value). For the preceding excited states, three short‐lived levels of different energy could be distinguished, too, similar to us and the Stolow group.

#### 4.3.2 The Stereospecificity of Cyclobutene ring Opening Depends on the Initial State and on Torsional Flexibility

The proposal that acceleration on going around the preceding CoIn determines the direction of passing through the last Coin can also be applied to ring opening of cyclobutene derivatives. This example is remarkable:

Since the beginning, the applicability of the Woodward‐Hoffmann (WH) rules has been explained with the paradigm of electrocyclic ring opening of cyclobutenes to dienes and the corresponding ring closure of dienes to cyclobutenes. For the ground‐state process, the WH rules predict conrotatory stereospecificity, preserving a C_2_ axis. From the excited state, the process should be disrotatory, preserving mirror symmetry. From the diene side, this is in fact observed, besides some cis‐trans isomerization. From the cyclobutene side, however, strong deviations were discovered mainly by Leigh and coworkers [109, 110, 111, 112, 113], a disconcerting situation in view of the role as prototype.

Thus, on ππ* excitation of a cis‐disubstituted cyclobtene one observes not only the WH‐allowed cis,cis‐dienes and trans,trans‐dienes (ccD and ttD) but also substantial quantities of the WH forbidden cis,trans‐dienes (ctD). The anti‐WH products were reduced on limiting the torsional flexibility by integrating the double bond into an additional small ring. On the other hand, even a perfect anti‐WH stereospecificity was observed, if instead of the ππ* state a Rydberg (π3s) state is excited [113]. (In cyclobutenes, alkylated at the double bond, the latter transition precedes the former). The contrast of this varying stereospecificity of ring opening with the perfect WH conformity of the corresponding ring closure seems to exclude a common pericyclic minimum (p*).

This dilemma was solved by the observation [114] that not only p* is in common but also a minimum (ct*), which is followed by the cis,trans CoIn (Figure 7). (Evidence was that for each minimum the time constant for leaving was the same, whether the wave packet came from the diene or the cyclobutene side [114]. The investigated isomer pairs were 1,3‐cycloheptadiene/bicycloheptene and 1,3‐cyclooctadiene/bicyclooctene). From the CB side, anti‐WH products form via ct*, WH products via p* [17, 114].

**FIGURE 7:**
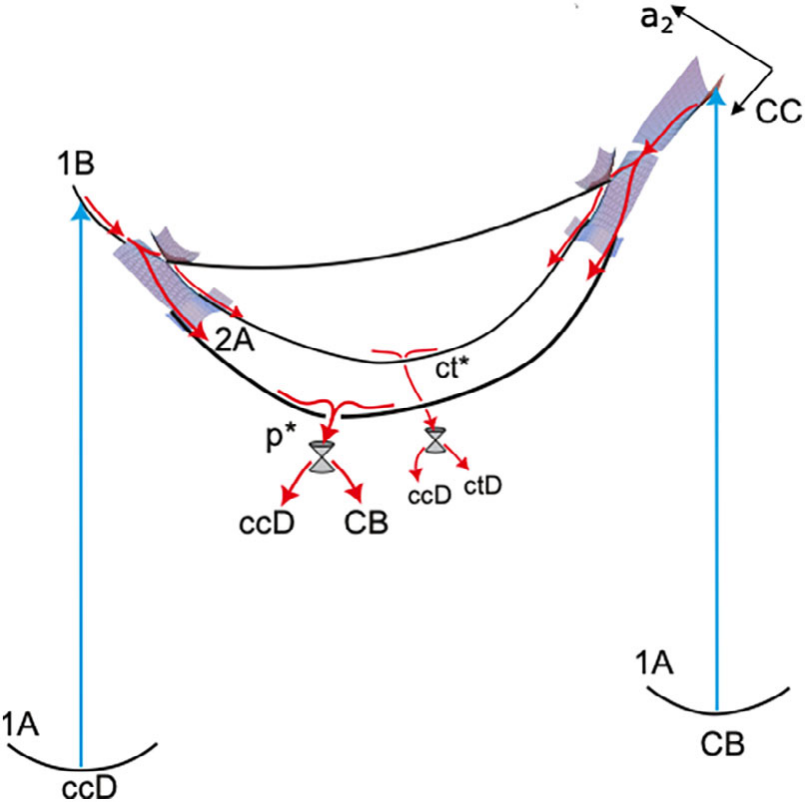
From both, a cis,cis‐diene (ccD) and the corresponding cyclobutene isomer (CB) a common pericyclic minimum (*p* ^*^) is accessible. The corresponding path involves—after the Franck‐Condon region, dominated by CC stretching—a disrotatory deformation. But also the minimum ct* is accessible from both sides; it is responsible for cis‐trans isomerization from the diene side, forming a cis,trans‐diene, ctD, that is an anti‐WH product from the CB side. This path is energetically (slightly) disfavored from the CB side. But this drawback can be compensated by a conrotatory (a_2_) momentum acquired in the Franck‐Condon region. An a_2_ momentum arises from C=C torsion in the ππ* state of CB; an additional one (with pyramidalization, see Figures 5 and 6), if the wave packet comes from the Rydberg (π3s) state and goes around the π3s/ππ* CoIn. In the latter case, the ct* path can be taken exclusively, leading to perfect anti‐WH specificity. From [114] (modified), with kind permission of Wiley.

The conrotatory path from CB (leading to ct* and anti‐WH products) is energetically less favorable than the disrotatory one. But the energetic difference is apparently small enough that it can be overcome by a momentum acquired in the Franck–Condon (FC) region (see the discussion in [114]). The energetic similarity of the two paths and their accessibility also from the CB side has to do with a geometric similarity [17, 114]: From the diene side, ring closure involves disrotatory torsion of the two conjugated double bonds, whereas Hula‐twist cis‐trans isomerization uses disrotatory torsion of one double bond and the central single bond. (Time‐resolved X‐ray diffraction of cyclooctadiene [115] has not yet the resolution to check this similarity). The two paths branch only after leaving the FC region of CB. This seems plausible, because the WH interactions can only begin after some nonplanar distortion that enables interaction of σ and π orbitals. (Temporal separation of FC slopes and reactive forces is also emphasized in Section 5.4).

A conrotatory (a_2_) momentum is generated by double‐bond torsion; resonance Rama spectra confirm that this vibration is FC active [114, 116]. This explains [114] (1) the observation of anti‐WH products and (2) the reduction of such products, if this torsion is hindered by fusion with rings of small sizes.

If the Rydberg (π3s) state is excited, surrounding the π3s/ππ* CoIn generates an additional a_2_ momentum by the slope toward the NADC vector (Figures 5 and 6), as pointed out in [17]. This is apparently sufficient for the complete anti‐WH stereospecificity [113] in this case [17].

To summarize, the deviation from disrotatory stereospecificity on CB ring opening is caused by a conrotatory (a_2_) momentum acquired in the FC region, before the WH interactions begin. It is C=C torsion on ππ* excitation. An additional a_2_ deformation arises, if a Rydberg (π3s) state is excited; it is caused by deflection in NADC direction on going around the π3s/ππ* CoIn. (Note that also here, the presence of a CoIn alone is not sufficient for explaining the dependence on the initial state: the acceleration is due to a slope). For the momentum mechanism, a prerequisite is that the paths are energetically not too dissimilar; otherwise, the wave packet would be deflected. For the diene/CB system, quantum chemical calculations exist, from where one can infer an energetic similarity of the paths of Figure 7 (see the discussion in [114]).

Similar calculations on the relative energetics for simple alkenes are still desirable (Section 4.3). In this case, Rydberg excitation enhances carbene formation (by hydrogen or alkyl 1,2‐migration). Such reactions (accompanied by ring splitting) are also observed with cyclobutenes, including enhancement by Rydberg excitation. A plausibility consideration in [17] suggests that 1,2‐migration is promoted, if the wave packet arrives at the last CoIn (or IS) with an additional momentum in direction of pyramidalization.

### 4.4 Highly Strained Photoproducts Are Formed via Strain‐Free CoIns Followed by Steep Exits

Many photochemical reactions give rise to highly strained products. From the excited state, the molecule reaches a branching region (usually the last CoIn), which also offers at least one path with practically no strain, namely the path back to the reactant (internal conversion). Then steep slopes below the CoIn must exist, so that the wave packet cannot return and revise an energetically unfavorable choice. The strain can only develop after the steep region. That is, the last CoIn must be practically strain‐free. This rule, formulated with cyclohexene cis‐trans isomerization as an example [24], has not found much attention, although it can help to guess the structure at a CoIn and help to understand formation of strained products.

Indeed, one of the cyclohexene photoproducts is a highly strained trans‐like isomer, which is very reactive and can be scavenged by methanol, e.g., [77, 117]. To find a possible CoIn for its formation, one can remember that in non‐cyclic alkenes the hydrogen or alkyl 1,2‐shift takes place only after twisting the double bond by 90°. In fact, it is possible to draw for the alkyl 1,2‐shift (forming cyclopentyl carbene) a structure with an alkyl bridge over a twisted C=C bond, which has no strain in the backbone (Scheme 2). Instead of completing the alkyl shift toward the carbene, we suggested that this shift can also be reversed and the twist be continued instead; only in this last phase the strain would develop [24].

**SCHEME 2:**
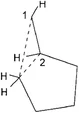
Suggested [24] strain‐free structure at the CoIn, from where both, cis‐trans isomerization and formation of cyclopentyl‐carbene can take place. Carbon atoms 2–6 are nearly in one plane. Migrating is the CH_2_ group containing C_6_. C_1_–C_2_ is the previous double bond, twisted by about 90°. The bridge (broken lines) forms a 3‐electron‐3‐center bond. Reproduced from [24] with kind permission of ACS.

In order that the strained product actually forms, one must also postulate that the wave packet cannot easily go around the lower cone but meets a high barrier there. Otherwise, the reaction would only be attempted but actually end in internal conversion. An example of the latter case is suggested in Section 5.5: On shorter‐wavelength excitation of cyclohexadiene, a higher‐lying part of the IS is accessible, corresponding to beginning (Hula‐twist) cis‐trans isomerization; apparently due to the developing strain, after passing the intersection the wave packet returns to the path toward the reactant (frustrated isomerization).

Indeed, the calculations of Wilsey and Houk for cyclohexene found just the structure of Scheme 2 for the alkyl shift CoIn (Figure 3b in [105]), although they did not mention that it can also be responsible for the trans‐like isomer.

In this example, the conclusion on the driving forces (slopes) and the CoIn is again based on the end products.

## 5 Cyclohexadiene ring Opening

### 5.1 Basics

The photochemical ring opening of 1,3‐cyclohexadiene (CHD) to *Z*‐hexatriene (formula scheme in Section 5.4.1) is a prototype of light‐induced electrocyclic reactions. Over the last decades it has been intensively investigated by quantum chemistry and time‐resolved experiments. Due to the tremendous progress in both fields, it is now one of the best understood reactions, regarding how it proceeds on the potentials. The review of Deb and Weber [21] focuses on CHD itself in the gas phase, whereas that of Arruda and Sension [118] also reports on derivatives such as 7‐dehydrocholesterol (precursor of previtamin D) and photochromic materials. The review of Ischenko and coworkers [12] also covers CHD ring opening, with some emphasis on time‐resolved diffraction experiments.

Based on quantum chemistry and some information from time‐resolved experiments, and described in terms of driving forces (slopes), the reaction proceeds as follows (with CHD as example [21]): After excitation to the lowest ππ* state (1B state), the molecule is first accelerated toward FC active coordinates. Due to the π antibonding, these also comprise (*C*_2_ symmetric) C=C twist, which in turn distorts the molecule, so that σ bonds (specifically the CH_2_—CH_2_ bond) can better interact with the π system. This activates the Woodward‐Hoffmann interactions. They cause a further downward slope along a *C*_2_ symmetric (“conrotatory,” ring opening) coordinate, till a minimum of the 1B potential. There it is crossed by the potential of a two‐electron excited state (π*^2^, 2A), which leads further down along the same direction to the 2A (“pericyclic”) minimum. (It results from the avoided crossing of the two correlation lines, connecting the π^2^ state of the reactant/product with the π*^2^ state of the product/reactant, as suggested by van der Lugt and Oosterhoff [1]). This excited‐state minimum is in common for forward (ring opening) and backward (ring closing) reaction (experimentally demonstrated in [17, 114] for the cyclobutene/butadiene system).

From the 2A minimum, a path which breaks the molecular *C*_2_ symmetry leads through a CoIn (actually two symmetry‐equivalent CoIns) to the ground state (1A), either back to the reactant or to the initial product *cis*,*Z*,*cis*‐hexatriene. Calculations predict a structure at the CoIn with C_1_ (one of the two CH_2_ groups, numbering as in hexatriene) is above C_6_ and C_5_, forming a three‐electron three–center bond, with the fourth electron delocalized on the remaining π system; see, e.g., the CoIn structures calculated by de Vivie‐Riedle, Robb, and coworkers [18]. Note that in another 3‐electron 3‐center system, triangular H_3_, the electronic state is degenerate, with the two degeneracy‐lifting coordinates: the removal of one H perpendicularly or in parallel to the remaining H_2_ group, respectively, as predicted by the Jahn–Teller effect. To reach the CoIn, the molecule gives the impression of in‐spiraling, as if it wants to reduce the ring size (Section 5.2). Deb and Weber hope for future evidence for a transient five‐membered ring [21]. For a time‐resolved diffraction method, detection of the 3‐electron 3‐center structure would be a too challenging task, due to the extremely short dwelling time of the molecule in the CoIn. But one can take the observed coherent oscillation as a support (Section 5.2). It is also worth noting that among the products of 1,2‐dihydronaphthalene (a CHD derivative) there was also the ring‐contracted benzo‐bicyclo [3,1,0]hexene, containing a five‐membered ring [119].

### 5.2 The Molecular Symmetry Is Broken Already on Passing by the 1B/2A CoIn, the Wave Packet Stays Compact and Passes Through a Localized CoIn to S_0_

A support for just this in‐spiraling is the coherent oscillation (ν_3_ in Figure 8) observed in our time‐resolved spectroscopy of cyclohexadiene in the region just before the last CoIn: We could assign it to a vibration along the antisymmetric coordinate that also connects the two symmetry‐equivalent CoIns [11, 21]. Such a vibration cannot be stimulated on the way from the 2A minimum to the last CoIn, because this region is very flat according to calculation [14]. But the wave packet meets a suitable slope on surrounding the first (1B/2A) CoIn [11, 14]; the wave packet is accelerated in direction of the nonadiabatic coupling vector (Figure 8). Hence the observed coherent oscillation infers the geometrical direction of a slope.

**FIGURE 8:**
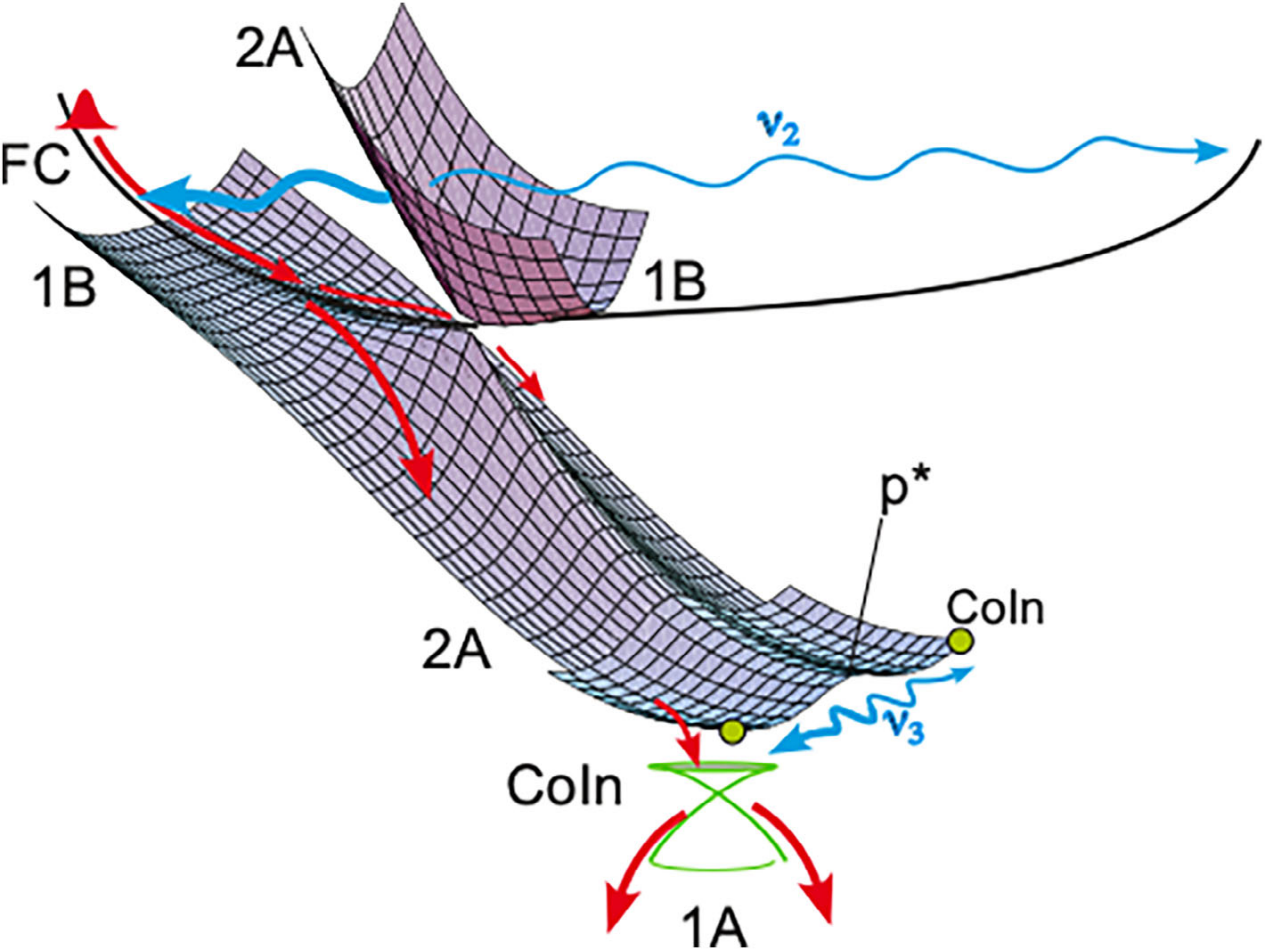
After leaving the Franck–Condon (FC) region of cyclohexadiene, the wave packet is deflected on passing by the first (1B/2A) CoIn. The deflection breaks the molecular symmetry, accelerates the molecule toward the last (2A/1A) CoIn, and stimulates the coherent oscillation ν_3_. (The figure is similar as in [120]).

The early acceleration—at the cusp of the first CoIn—also answers the question, how the molecule can reach from the 2A surface the last CoIn in such a short time (80 fs), although the CoIn entrance is calculated to be very flat. The question seemed even more serious, when Robb and coworkers calculated that this is not a local problem: the minimum energy path (MEP) on 2A is largely parallel to the MEP in the IS [18]; that is, there is no downward slope toward the IS. One can also conclude that—due to momentum effects—the molecule does not follow the minimum‐energy path.

This IS‐MEP [18] also comprises a kink CoIn (Hula–twist CoIn) (already found by Tamura [121]), accessible at higher energies, which would be responsible for cis‐trans isomerization in less constrained systems, but in the given case can only lead back to CHD (internal conversion). But on considering energies, slopes and the measured ring‐opening yield, the authors suggest that this channel plays a minor role at most [18] on excitation of the 1B state (see, however, Section 5.5 for excitation of a higher state).

Higher‐level calculations, for example those of Pemberton and coworkers [122] using MS‐CASPT2, point out—consistent with our results—the central role of the 2A state; it gives rise to the pericyclic minimum and contains about 50% of two‐electron excitation, the rest consisting in one‐electron excitation involving orbitals different from HOMO and LUMO. Schalk, Tapavicza and coworkers [123] suggest that such an intermediate excited state is not necessary, on the basis of time‐resolved density functional calculations (which is not able to handle two‐electron excited states). They point instead to the character of S_1_ changing along the relaxation path. The changing molecular (and state) symmetry is not mentioned. It is hard to see how they would explain our observation of an oscillation along a symmetry‐breaking coordinate.

The pericyclic (2A) minimum was also identified by X‐ray spectroscopy [124] (see Section 5.3).

Schalk and coworkers [123] also report on two sets of trajectories: a fast one giving rise to ring opening, and a slow one leading back to CHD; they suggest to assign two observed time constants to these “parallel processes.” However, parallel reactions would need two strictly separated precursors; a branched reaction (one precursor, two products) would exhibit only one time constant, the lifetime of the precursor. It is not obvious what kind of a barrier in S_1_ could separate the two sets of trajectories. The Tapavicza group already presented similar calculations on the ring opening in dehydrocholesterol, with separate productive and unproductive trajectories [125], as mentioned in Section 5.4.

We can also say that the region, where the wave packet passes through the IS toward hexatriene, is not extended but rather localized. This can be inferred from our observation that the wave packet needs only 20 fs to pass through a region on the ground state surface, where the ionizing laser meets a resonance in the hexatriene ion [126]; this means that the wave packet is still compact after passing the CoIn, hence not an extended region of the IS [126]. A persisting compactness of the wave packet is also implied by the observation of a coherent oscillation in S_0_ (probably in the product *Z*‐hexatriene) by the Suzuki group [127]. This contradicts to some calculations, which suggest that the wave packet diffuses on its way to fill the full phase space [121, 128]. (The latter paper even suggested that therefore also *E*‐hexatriene may be among the products [128], probably an artifact of their simplified potentials). It was previously pointed out that the path down the potentials is not a conventional statistical relaxation but a coherent process, as can already be concluded from the observed coherent oscillations [83].

However, the main idea of Schönborn, Sielk, and Hardtke [128] fits nicely: They assumed a localized leak from S_1_ (the S_1_/S_0_ CoIn) and described in this way that the ring is opened (or S_1_ is depopulated) in a stepwise manner, synchronously with the oscillation observed by us in S_1_. The idea was recently taken over [120] and compared with similar phenomena in photodissociation of metal carbonyls (Section 7).

It is also worth noting that the observation of a ring vibration (the antisymmetric in‐spiraling) in the region before the last CoIn means that the ring opening is not completed before passing through this CoIn, although it is already weakened in the excited states [11]. In diffraction experiments—and even in calculations—it is not so easy to decide, when exactly (or where on the potentials) the ring is definitively opened. On the other hand, by a combination of time‐resolved electron diffraction and quantum‐chemical calculations, Jiang and coworkers saw indications of rehybridization in the pericyclic (2A) minimum [129].

### 5.3 Cyclohexadiene as a Prototype

The cyclohexadiene case is not only a prototype for derivatives, as described in the Arruda–Sension review [118] but also for electrocyclic reactions involving larger or smaller rings. The symmetry‐breaking displacement of the last CoIn is again the seeming transient ring contraction. Such intermediate structures played a role for explaining the varying stereoselectivity in cyclobutene ring opening (Section 4.3.2).

Section 5.4.2 will present two more examples of electrocyclic reactions (cyclopentadiene and 1,3‐cyclooctadiene), which were also studied by time‐resolved diffraction.

The van der Lugt–Oosterhoff scheme (with the initial one‐electron excitation and the following take‐over by a two‐electron excited pericyclic minimum [1]) is more generally valid for all pericyclic photochemical reactions. But the displacement of the last CoIn must be calculated individually for each reaction type. An early review of pericyclic reactions investigated by time‐resolved spectroscopy can be found in [130].

Because the cyclohexadiene ring opening has been so well investigated, it serves also for testing experimental and theoretical methods. Of special interest in our context are diffraction experiments, because there is hope that they can elucidate, how the molecule evolves geometrically during the reaction. In the case of X‐ray scattering the necessary time resolution can be reached; however, the low numbers of photons scattered from isolated molecules remains challenging [9]. Not many details were revealed in this experiment in 2015. But critical progress was obtained already in this work [9] by using an X‐ray free‐electron laser and even more by a work from the same group [131], in which the ring‐opening quantum yield (after excitation of a higher state) and time constants (in the hot ground state) could be determined with good signal‐to‐noise ratio. Section 5.5 will further discuss this experiment.

Due to the higher scattering cross section, electron diffraction provides much more signal. However, space charge deteriorates the time resolution. Much progress in this respect was obtained by using relativistic electrons (resolution 160 fs [8] or recently even 80 fs by using a dispersive focusing system [10]), and the method was tested with cyclohexadiene [8]. Diffraction from isolated molecules reveals the probabilities of distances of atom pairs; it is difficult to extract direct information on the direction of motion. Therefore (and for further interpretation of the data) diffraction experiments are accompanied by quantum‐mechanical calculation of the wave packet path. For example, there was no information, where—and in which direction—the molecular symmetry is broken, in contrast to the conclusions above (Section 5.2). The data (and the calculation) also give the impression that the CH_2_–CH_2_ σ bond is already stretched from the beginning (on π → π* excitation), whereas we found evidence (Section 5.4) that it begins only with delay (our better time resolution of 13 fs was helpful) and is initiated by a C=C bond twist (which was not detected in the diffraction experiment). Nevertheless, in view of the progress in the last years, one can expect that diffraction methods will in future reveal more details of the dynamics.

Another example is experimentally less intricate: it used time‐resolved near‐edge X‐ray absorption [124]. It detects the absorption from 1s (core) orbitals to valence orbitals and thus finds their occupation and energy. In the given case, it detected, e.g. the pericyclic (2A) minimum and the time for its population (60 fs). It also found a hexatriene/cyclohexadiene branching ratio at the last CoIn near 60:40, nearly the same as in solution. (A similar branching ratio in the gas phase was also found by the Suzuki group [132] in photoelectron spectroscopy by 14 eV photons, which revealed also the ground stater of the products). Deb and Weber suspected (in 2011) that in the gas phase, hexatriene is formed with near 100% quantum yield [21] (see also the discussion by Arruda and Sension [118]).

Recently the Suzuki group applied their time‐resolved photoelectron spectroscopy with 21.7 eV photons for ionization also to cyclohexadiene [127]. They analyzed and simulated their intermediate spectra in detail. The lifetime of the spectroscopic state (= sum of the first two time constants, 31 fs; time resolution 48 fs) was about the same as found previously. However, the two‐electron excited (2A) state was found to decay already within 37 fs (previous value around 80 fs [11]). In the ground state they found a coherent oscillation, probably taking place in the product hexatriene. This means that, in spite of passing before through two CoIns and other anharmonic regions, the wave packet is still compact, consistent with our finding mentioned at the end of Section 5.2. A coherent oscillation in S_0_—but this time in the reactant CHD derivative α‐phellandrene (after Rydberg excitation)—was also found recently [133].

Another recent work pointed out that the 2A pericyclic minimum correlates at earlier geometries with a higher two‐electron excited state (3A^–^) [134]. This work was combined with time‐resolved photoelectron spectroscopy, using a free‐electron laser (photon energy 19 eV) as a probe; time resolution was limited by the time steps of 100 fs.

The Barbatti group [135] further developed (and critically compared with previous approaches) the quantum chemical description (with balanced treatment of electronic structure), focusing on the description of S_2_, S_1_, and S_0_ surfaces and their intersections, combined with surface hopping, and the results compared with experimental observations (including the quantum yields and lifetimes) and previous theoretical results. Among other things, they pointed out that the dark 2A state is really involved (which was doubted in time‐resolved density functional calculations). They also report on an extended IS (see, however, Section 5.2). Salazar and Faraji compared spin‐flip density calculations for the electrocyclic reaction with such higher level calculations, with encouraging results [136].

Ohta and coworkers calculated the CHD ring opening by a surface hopping full‐dimensional model, using potentials on the MS‐MR‐CASPT2 level [137]. Many results and their general description were consistent with previous calculations, although their excited‐state lifetimes are a little short, compared with measurements. Remarkably for trajectory methods, they also find recurrences, which agree well with our coherent oscillations, also in their assignments. They also consider direct excitation of the 2A state and predict that the hexatriene quantum yields are smaller than with 1B excitation. They try to find a correlation of the quantum yields with the exact location, where the trajectories pass to the ground state. (Compare our interpretations of wavelength dependence in Sections 4.3 and 5.5, which uses momentum directions instead of locations).

Lei and coworkers tested their on‐the‐fly calculation method successfully with cyclohexadiene ring opening [138]. They only used computed potentials and gradients, combined with surface hopping.

### 5.4 The Woodward–Hoffmann Interactions Are Selected and Activated Only With Delay

Deb and Weber [21] collected also sources of information on the initial dynamics, i.e., in the FC region. Among others, it came from the vibrational structure in their time‐resolved photoelectron spectra, from the coherent oscillations in our multiphoton ionization signals and from resonance Raman spectra.

Whereas we only see activity of CC stretch and a twist (ring puckering) vibrations, the resonance Raman spectra also show CH_2_—CH_2_ stretch and (conrotatory) twist. These and other delocalized vibrations can be rationalized, assuming stimulation by one local driving force: the π antibonding nature in the 1B (ππ*) state changes bond lengths and increases twist angles in the π system. The latter must be *C*_2_ symmetric (conrotatory), because the antisymmetric (disrotatory) one is not FC active in the *C*_2_ symmetric molecule.

After some excursion, the FC forces would come to an end, and the molecule would reach a displaced minimum. However, after the C=C twist has caused sufficient extension and erection of the CH_2_—CH_2_ bond, it can interact with the π system, and from this point on, the Woodward–Hoffmann (WH) interactions “are switched on” and provide an additional downward slope, eventually leading to conrotatory ring opening.

For a long time, it was believed that the WH rules would be active from the beginning, right after excitation. But how would σ and π orbitals interact in cyclobutene before it is distorted out from its planar structure (Section 4.3.2)? And how would cyclohexadiene decide between (conrotatory) ring opening to *Z*‐hexatriene and (disrotatory) ring closure to bicyclo‐hexene, which are both WH allowed? The answer in this case: The former proceeds from the chair‐like (*C*_2_ symmetric) conformer, the latter from the boat‐like (*C*_s_ symmetric) conformer [139]. FC active coordinates are totally symmetric, i.e., *C*_2_ or *C*_s_ symmetric, respectively. After excitation, the molecule would first move in the individual direction of FC activity and thus select, which WH case applies. The conformations may result from thermal equilibrium or may be due to integration in a ring system or caused by the environment. This kind of conformer control is reviewed and explained and further developed in [17]. Section 5.4.1 develops this mechanism further.

The concept of two separate sources of downward slopes (separate on the potential or in time) provides also an explanation, why some derivatives of cyclohexadiene (such as the polycyclic 7‐dehydrocholesterol) can fluoresce, at cryogenic temperatures even with good quantum yield. Arruda and Sension compiled a number of examples [118]. Obviously, these molecules need a (minor) activation energy to leave the FC region (for dehydrocholesterol around 2 kJ/mol, as to conclude from the temperature dependence [118]). This is plausible, if the C=C twist is hindered and needs additional energy to reach an excursion sufficient to activate the WH interactions. We already suggested this in [11].

It seems that this initial, activated process is rate‐limiting for the ring opening in dehydrocholesterol; that is, leaving the 2A surface is faster than leaving the initial 1B state. This can be inferred from the 1B lifetime (estimated from the fluorescence yield: 1–2 ps), whereas excited state absorption showed a doubly exponential decay with 0.6 and 1.9 ps [118, 140]) and the appearance of the product within about the same time (1.5–2 ps, measured by time‐resolved circular dichroism [73], see Figure 2). The doubly exponential decay is consistent with this model, with 1B and 2A lifetimes 1.9 and 0.6 ps, respectively. The observations are not consistent with a branched process from a single species, decaying in a fast and a slow channel (rates *k*_1_ and *k*_2_): There would be only a single lifetime for this species, *τ *= (*k*_1 _+ *k*_2_)^–1^. The branched process was initially favored in [118], based on calculations of Tapavicza and coworkers [125] with only one excited state but two sets of trajectories, one leading to ring opening, the other back to the reactant.

The more recent experiments of the Sension group [140] confirm the time constants, using different pump wavelengths. They interpret their observations on the basis of calculations with massive computer power [141]. This calculation involves (as many previous works on cyclohexadiene) the two states 1B and 2A, which respectively form the upper and lower part of S_1_, the trajectories passing by a 1B/2A CoIn. It also finds two time constants, which the authors attribute to “nonequilibrium dynamics.” The calculated decay is initially fast and then slower. But the fluorescence must clearly be assigned to the FC region. Because it has a lifetime of 1–2 ps, the longer time constant must belong to the earlier region and the faster process is second. It would also be interesting to see in theory and experiment, whether the longer one of the time constants depends on temperature, like in an activated process that is suggested here.

The idea that acceleration along FC active coordinates precedes reactive interactions was proposed already in 2001 [14], also as a basis for interpretation of the principle of least motion (Section 5.4.1). It was observed there [14] that in a 1,4‐dialkyl substituted CHD (α‐terpinene) the traveling time through the FC region was prolonged (from 10 to 40 fs), whereas the other regions were not much affected; 2,5‐disubstitution (α‐phellandrene) had not much effect in any region. Slowing down of the motion in the FC region could be readily attributed to a mass effect on the FC‐active torsion. In agreement with these findings, a high‐level calculation also found that 1,4‐disubstitution slows down the (total time for the) ring opening, whereas 2,3‐disubstitution does not [142]. However, for interpretation, the authors focus on the time for breaking the saturated C—C bond (where the experiment [14] found nearly no effect).

#### 5.4.1 Acceleration Toward Coordinates, Made FC Active by Predistortion

The following briefly explains the ideas [17] how in a molecule, such as cyclohexadiene, distortion in the ground state makes the distortion coordinate FC active and how the minor energy differences in S_0_ can be magnified in S_1_ (Figure 9).

**FIGURE 9:**
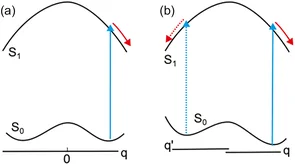
Acceleration in the excited state into the predistortion direction (*q*) in the case that *q* is antisymmetric. (a) and (b) show the cases of symmetry‐equivalent and non‐equivalent conformers. In (b), *q*’ can either be = –*q* or be a different coordinate (for example in cyclohexadiene, *q*’ can be disrotatory and q conrotatory). From [17] (modified), with kind permission of Elsevier.

If cyclohexadiene were planar (*C*_2v_ symmetry), the two C=C torsions would be antisymmetric (a_2_ and b_1_) and hence not be FC active (horizontal slope in S_0_ and S_1_ in Figure 9). A symmetry‐breaking distortion (in this case by internal forces) in one of these directions (*q*) results in either C_2_ or C_s_ symmetry (chair‐like or boat‐like conformations, respectively) with symmetry‐equivalent minima (Figure 9a). Thereby, the deformation coordinate becomes FC active, that is, a slope appears in the excited state.

Figure 9a applies to symmetry‐equivalent conformers; with cyclohexadiene, *q* can either be a conrotatory or a disrotatory deformation. Obviously, after excitation the molecules are accelerated into the direction of predistortion. The figure hence explains, why chair‐like cyclohexenes open their ring on excitation, whereas boat‐like cyclohexenes undergo ring closing to bicyclic cyclobutenes [17] (Scheme 3). This rule was only empirical before [139]; the review of Dauben [139] has compiled very many examples.

**SCHEME 3:**
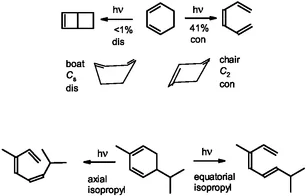
Different photoproducts result from different conformers. The shown paths are all Woodward–Hoffmann allowed. The initial distortion decides on the choice between disrotatary and conrotatory or between clockwise and counter‐clockwise.

The environment or substituents can make the conformers inequivalent (Figure 9b). An example with substituents is α‐phellandrene, a cyclohexadiene derivative with a methyl group at one of the double bonds and an iso‐propyl group attached to one of the CH_2_ groups (Scheme 3); in one conformer, the bulky substituent is equatorial (*eq*), in the less stable one axial. The WH rules allow both clockwise or counter‐clockwise double‐bond twisting in conrotatory ring opening, which produces either an *E*,*Z*‐triene or a *Z*,*Z*‐triene. Hence, the WH rules would allow both products from each conformer. But Figure 9b explains that the molecule prefers the direction of predistortion, as if it followed a “principle of least motion”; also this principle [143] and the “accordancy principle” [139] were only empirical before. Many more examples are presented in [139]. In [17], the amplified predistortion is invoked to interpret them, as well as other forms of conformer control (such as chiral induction) and several momentum effects.

Champenois and coworkers recently confirmed by time‐resolved electron diffraction with accompanying trajectory calculations that also in the gas phase the *eq* conformer of α‐phellandrene produces the *E*,*Z*‐triene [144].

The principles of amplified predistortion and of delayed choice between several allowed reactions are more general [17]. Figure 9 can also be applied to cis‐trans isomerizations, for example (such as used for molecular motors). Also a quantum‐chemical calculation confirms that the momentum acquired in the FC region dominates the choice between possible reactions:

The Gordon group found by spin‐flip density functional theory for cis‐stilbene (which is distorted from planarity) a relatively flat excited‐state landscape [145]. Whereas the initial small slope points toward cis‐trans isomerization (the direction of predistortion), the steepest slope then turns toward ring‐closure to dihydrophenanthrene. In the trajectory calculations, due to their initial momentum the wave packets nevertheless prefer their initial direction toward the cis‐trans CoIn.

It might be suspected that predistortion, e.g., by a matrix only hinders competing reactions, because: How can a matrix accelerate anything? However, an amazing example demonstrates that distortion actually can act as acceleration: Compared with solution, in the opsin matrix the cis‐trans isomerization of the chromophore of rhodopsin is (1) much faster, (2) has a higher quantum yield, and (3) is highly selective for a single double bond. These observations are for the first time rationalized by the acceleration along predistortion coordinates [17].

Section 5.4.2 explains that instead of down‐slopes in the FC region (as in Figure 9) a flattening of the potentials can be sufficient.

#### 5.4.2 Cyclopentadiene and 1,3‐Cyclooctadiene

The idea that the FC region precedes reactive interactions can also be assumed in two more examples: cyclopentadiene and 1,3‐cyclooctatriene. Both undergo electrocyclic ring closure to cyclobutene derivatives, besides other photoreactions. They were recently also investigated by time‐resolved diffraction, accompanied by simulations [115, 146, 147, 148]. These diffraction works also give rise to a minor modification of our previous assignments for cyclopentadiene [16, 17]; for cyclooctadiene, conformers are now taken into account below.

Cyclopentadiene shows three photochemical channels [16, 17]: a sigmatropic 1,3‐H‐shift, a disrotatory and a conrotatory electrocyclic ring closure to bicyclopentene (BP) and tricyclopentane (TP), respectively (Scheme 4). (Ring opening products, reported by the diffraction experiments [147, 148], are possibly formed from the hot ground state of CP). The molecule is initially planar. Therefore disrotatory (*C*_s_) and conrotatory (*C*_2_) double‐bond torsions seem to be equally active (in second order) in the FC region. Disrotatory deformation also lifts the CH_2_ group out of plane, so that the migrating H atom approaches to the accepting C_2_ or C_3_. Hence, this motion prepares not only Bp formation but also H migration.

**SCHEME 4:**
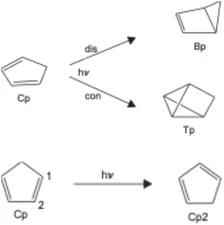
Photoreactions of cyclopentadiene. (Cp) Whereas the disrotatory reaction to bicyclpentene (Bp) is expected to be concerted, the conrotatory path to tricyclopentane (Tp) is not necessarily synchronous. The 1,3‐sigmatropic hydrogen migration (bottom) was postulated on the basis of the deuterium isotope effect [16].

By contrast, in the conrotatory direction, C_1_ and C_4_ move to opposite sides of the plane, a deformation that is soon hindered by the bridging CH_2_ group. Probably the corresponding barrier emerges early on this path so that the wave packet still can return and take the disrotatory direction with higher probability, without fully suppressing the other path.

It should be realized that the FC activity is dominated by bond‐length changes in the conjugated system, whereas the torsions are inactive in first order; only the force constant is reduced, even if strongly. Hence, torsional wave packets can still freely oscillate and thus return from the “wrong” (conrotatory, hindered) direction. The reduced force constant allows for spreading of the wave packets and for exciting higher vibrational states (in transitions with even Δ*v*).

In fact, in the diffraction experiments [147, 148], Bp was the main photoproduct, Tp was at the detection limit, and the Tp:Bp yield ratio was calculated [148] to 1:20. Ring opening products were also barely detectable. The diffraction experiments are not suitable to detect H migration. Our evidence [16] for it was the strong deuterium effect on the time constant *τ*_3_.

The X‐ray diffraction [147] also revealed time constants. Their shortest one (88 fs) was for the rise time of Bp. It coincides (within the limits of their time resolution) with our *τ*_3_ = 71 fs, the time constant that we assigned to H migration on the basis of the deuterium effect. This is evidence that both reactions originate from a common branching point; *τ*_3_ is then the lifetime of this precursor, and this lifetime is dominated by the fast H migration.

Therefore, the processes on the cyclopentadiene potentials are now revised (Figure 10): After leaving the FC region and the 1B surface (*τ*_1 _+ *τ*_2_ = 37 fs), two minima on the 2A surface are reached, displaced in disrotatory and conrotatory direction, respectively. From the (favored) former, the path branches and reaches within *τ*_3_ two close‐lying CoIns (or locations in the IS), responsible for H migration and electrocyclic Bp formation, respectively. From the less favored 2A minimum, displaced in conrotatory direction, the wave packet leaves within *τ*_4_ through a CoIn_con_ toward Tp; the latter path is indicated by broken lines in Figure 10. The lower probability of this path is reflected in the signal ratio of the locations L_3_ and L_4_. (Previously we assigned *τ*_3_ and *τ*_4_ to the H migration and the disrotatory ring closure; the conrotatory path was believed to be covered by the other signals).

**FIGURE 10:**
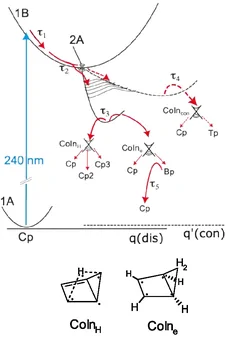
Potentials and wave packet paths for cyclopentadiene photoreactions (revised compared with [16]). The measured values for the time constants τ_1_–τ_5_ were 22, 15, 71, 333 fs and 19 ps, respectively. It is now assumed (1) that H migration and electrocyclic ring closure start from the same 2A minimum, both associated with the same time constant (τ_3_) and (2) that τ_4_ belongs to TP formation via a conrotatory path (indicated by broken lines), starting from a second 2A minimum and passing through separate CoIn (CoIn_con_). Branching of the disrotary and conrotatory paths must occur before, at the 1B/2A CoIn or probably even before. The formulas suggest structures for the H‐shift CoIn and disrotatory electrocyclic CoIn, showing their similarity; for the latter, breaking of the molecular *C*_s_ symmetry (in‐spiraling similar to cyclohexadiene) is not indicated.

In the X‐ray diffraction, ring opening products appear within 590 fs; the signal was weak, however. Our *τ*_5_ = 19 ps for ring opening of hot BP was also observed in the diffraction experiments [147] and the simulations [146] and the assignment confirmed.

In their simulations, Bertram, Weber and Kirrander [146] see no photochemical 1,3‐sigmatropic H shift but only a ground‐state hydrogen 1,2‐shift (that is, a WH allowed 1,5‐sigmatropic H migration). The latter process cannot be detected by our method, because there is no energy change; also elimination of an H atom (in the hot ground state) would elude observation. A calculation of the deuterium effect on the various time constants would be desirable. The 1,3‐sigmatropic H migration is not mentioned either by the previous calculations, including the RASSCF work of Santolini and coworkers [149]. But the latter work clearly shows the closeness of a CH_2_‐hydrogen to the C_2_ (atom numbers 3 and 6 in part f of their Figure 10), when the molecule has reached the region of the disrotatory S_1_/S_0_ CoIn (CoIn_e_).

In this [149] and the other calculations (e.g., [146, 150, 151]), non‐totally symmetric (mainly disrotatory) deformations of the potential begin in the region of the first (S_2_/S_1_, 1B/2A) CoIn. This is a support of what was said above: that in the FC region the potential is only flattened in these directions. But the flattening allows for the wave packets to spread out in disrotatory direction; in conrotatory direction, amplitude spreading is probably smaller due to hindering.

Kuhlman and coworkers [150] compare their calculations also with time constants from time‐resolved photoelectron spectroscopy: For S_1 _→ S_0_, they report 51 fs, shorter than our 71 fs [47]; the calculated time (28 fs) is even shorter.

Besides cyclopentadiene and 1,3‐cyclohexadiene, we also investigated two larger cyclic dienes: 1,3‐cyclohepatiene and 1,3‐cyclooctadiene [47]. They undergo mainly two photoreactions (Scheme 5): *cis*‐*trans* isomerization and electrocyclic ring closure to a cyclobutene [152, 153]. In contrast to the former, the latter reaction exhibits a strong dependence of quantum yield on the ring size [152, 153]. Leigh correlates it with the dihedral angle between the double bonds, presenting also more examples [152, 153, 154].

**SCHEME 5:**
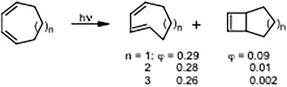
Cyclic cis,cis‐1,3‐dienes (ccD) with their photoproducts cis‐trans‐1,3‐dienes (ctD) and cyclobutenes (CB) and the corresponding quantum yields φ according to [152, 153]. From [17], with kind permission of Elsevier.

We could attribute two of the measured time constants to traveling times in two valleys of the 2A (S_1_) surface (Figure 11). The observation of two different time constants means that the valleys are separated by a barrier, which prevents rapid exchange of population. A support for the assignment was that the ratio of signal intensities from the *τ*_4_ valleys reflected the ratio of ring‐closure quantum yields for the two ring sizes. Another support comes from the recent calculation of Chakraborty and coworkers [155] (see below). Hence the two paths branch before, perhaps near the first CoIn or already in the Franck–Condon region (Figure 11).

**FIGURE 11:**
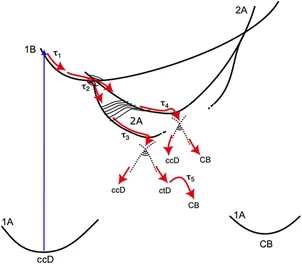
Suggested [47] potentials and wave packet paths for 1,3‐cyclo‐hehepadiene and 1,3‐cyclo‐octadiene. ccD and ctD are the cis,cis‐dienes and cis,trans‐dienes, CB the cyclobutene derivatives. The figure already indicates that both 2A minima are accessible also from the CB side. As explained in Section 4.3.2, this accessibility plays a crucial role for explaining the varying stereospecificity in cyclobutene ring opening. From [47], with kind permission of Taylor and Francis.

The calculations of trajectory surface hopping dynamics of Chakraborty and coworkers [155] not only found the products mentioned above but also some carbenes, analogous to those observed with alkenes (Section 4.3), resulting from 1,2‐H‐shift and from 1,2‐alkyl shift. Interestingly, these products are formed on a longer time scale than the others. This means, they cannot have a common immediate precursor (branching point) but must result from separate valleys on the S_1_ surface, analogous to those which we inferred from our experiment [47] (see above).

Importantly, they find two ground‐state conformers [155]. In the more abundant one (66%), the diene unit is *C*_2_ symmetric with a slight (conrotatory) torsion of the central bond, in the less abundant one (33%) the diene is *C*_s_ symmetric. This suggests that that perhaps the product ratio is conformer controlled and that the (disrotatory) ring closure starts only or mainly from the *C*_s_ conformer. So far, this idea was not considered in the interpretation of the strongly varying product yield in cyclic and exocyclic dienes [152, 153]. Instead, a correlation with the central‐bond torsion was invoked [153]. However, if the minor conformer alone were responsible for ring closure, the cyclobutene should form from it in substantial quantum yield. This is not the case with the conformationally (*C*_s_) fixed norbornane with two exocyclic methylene groups (cyclobutene quantum yield 2.5%) [152, 153]. It can therefore be assumed that both reasons (conformer control and central bond torsion) play a role. (It should be mentioned that the ring closure to these cyclobutenes occurs with high disrotatory stereospecificity [152, 153]. Surprisingly, Chakraborty and coworkers [155] show in their Supporting Information only a CoIn with conrotatory deformation).

It may be supposed that the different valleys on the 2A surface are selected already in the FC region. Such an assumption may explain why the predicted [155] carbenes are not formed: There is no predistortion that would direct the wave packet into the corresponding valleys.

On the basis of an early choice of direction one can also interpret why the yields of cis‐trans isomerization do not depend on ring size of the cyclic and exocyclic dienes, whereas those for ring closure strongly vary with it [153] (or with the dihedral angle at the central bond [152, 153]): Hula‐twist cis‐trans isomerization is triggered by an attractive 1,3‐interaction in the 2A state (section 3), which is active over a large range of dihedral angles. By contrast, a conrotatory predistortion (main conformer) disfavors the valley with disrotatory geometry, leading to cyclobutene ring closure.

The calculations [155], done in the Weinacht group, helped also to analyze the time‐resolved photoelectron spectra, measured in the same group [156]. They found an excited‐state lifetime around 50 fs, similar to our *τ*_3_. But they could not distinguish different observation windows in the various states or the reaction channels from there. Similar results were obtained, when the molecule was excited at shorter wavelength and the probe wavelength was varied [157]. In both works, the authors consider, why the calculated lifetimes are by a factor of 2 larger than measured.

A recent time‐resolved electron diffraction [115] (with participation of the Weinacht group) recognized the cis‐trans isomer and ring‐closure product. In the excited state, the diffraction revealed that the ring was more stretched and slimmer; the simulations confirmed it and attributed it to the deformation (bond lengths, torsion, and pyramidalization) of the diene part [115]. The experiment found an excited‐state lifetime around 74 fs, very well consistent with our traveling time [47] from the Franck–Condon region to the CoIn for cis‐trans isomerization of *τ*_1 _+ *τ*_2 _+ *τ*_3_ = 77 fs.

In order to find generalizations, the authors of the simulations [115, 155] reminded of the similar deformations in the excited states and CoIns of ethylene and alkenes, noting also that for many trajectories the distortions are localized to one of the two double bonds. Further insight is provided by consideration of driving forces:

Bond lengths begin to change already in the FC region; also torsions (in the pre‐distortion direction) are FC active. One‐sided pyramidalization in ethylene and alkenes (Sections 4.1 and 4.2) begins in the 2A state, because this two‐electron excited state is zwitterionic in ethylene and alkenes, and the negatively charged carbon prefers sp^3^ hybridization. This is frequently accompanied by 1,2‐migration of a hydrogen or alkyl group (forming carbenes), which is initiated at the 1B/2A intersection (Section 4.1). However, the lowest two‐electron excited state (2A) of dienes and polyenes is not zwitterionic but covalent [1]. Perhaps on localization of the deformation, a higher–zwitterionic–state comes close. To find out, it would help to project the molecular‐orbital wave functions to valence‐bond states. It may rationalize the pyramidalization at the outer (C_1_ or C_4_) carbons (at higher energy [155]). For the lower‐energy [155] inner pyramidalization (C_2_ or C_3_), a different mechanism (in the non‐polar 2A state) is suggestive: The 1,3‐attraction in Hula‐twist cis‐trans isomerization reduces the angle at C_2_ and thus induces its rehybridization (Section 3).

This section again underlines the idea that the initial choice of direction depends on the events in the FC region and that reactive interactions are switched on only with delay. FC activity in first order is not a necessity; it already helps, if the curvature (e.g., in the direction of torsion) is reduced.

A further remark on Section 5.4 may be worth noting: The concept of delayed activity of reactive forces may be considered a generalization of the idea of predissociation. Predissociation is normally explained by tunneling from a bound to a repulsive potential; it works also for diatomic molecules and it also preserves coherence. The delayed reactive forces are here described by a path around a CoIn (for polyatomics); it is applicable for many reactions, not only dissociation.

### 5.5 Wavelength Dependence of Cyclohexadiene

The Weber group investigated the dynamics of cyclohexadiene excited near 200 nm to 3p Rydberg states. In a first experiment with time‐resolved photoelectron spectroscopy [158], they found that from the Rydberg state the molecule returns to the ground state within little more than 200 fs, remarkably with substantially reduced yield of ring opening compared with excitation to the 1B state near 267 nm. In an experiment with time‐resolved X‐ray diffraction [131], this reduced yield was quantitatively determined to 24%, about half of that on 1B excitation. The experiment also showed clearly the equilibration between cyclohexadiene and hexatriene in the hot ground state in the time range of several hundred picoseconds.

By contrast, the Sekikawa group found in two experiments with two‐photon pumping at 400 nm [159, 160] that the reaction path leads via the 2A state and 2A/1A (S_1_/S_0_) CoIn with hexatriene quantum yields practically unchanged compared with 1B excitation. Their probe was photoelectron spectroscopy with a high‐harmonic in the first experiment and high‐harmonic generation by the molecule itself in the second. Due to their poor signal‐to‐noise ratio and due to uncertainties caused by two‐photon excitation, the results of the X‐ray diffraction [131] seem more reliable. In a third experiment, with two‐photon pumping and soft X‐ray absorption as a probe [161] the authors speak about a direct jump from the Rydberg state directly to S_0_, mainly to that of cyclohexadiene (instead of a continuous path over intermediate potentials and CoIns). A process between 400 and 600 fs was assigned to the ring opening in the hot ground state (the picosecond range was not investigated), nearly three orders of magnitude faster than in the diffraction experiment [131]. The latter is supported by the more direct evidence and again the better signal‐to‐noise ratio. Also in a fourth experiment, this time with the CHD derivative α‐phellandrene (formula in Scheme 3), the Sekikawa group interpret their results by a radiationless vertical ultrafast Rydberg → S_0_ relaxation, before a ring opening begins in the hot S_0_ [133].

To rationalize the lower ring‐opening quantum yield, Weber and coworkers [131] suggest that from the Rydberg state the wave packet finds its way (via intermediate potentials and CoIns) to a high‐lying branch of the 2A potential and from there directly through the 2A/1B CoIn to the C_2_‐symmetric S_1_/S_0_ CoIn (part of the IS). (By contrast, on 1B excitation the wave packet passes around the 2A/1B CoIn, with breaking of the molecular symmetry, to the CoIn_min_). In fact, a calculation of the Nanbu group [137] found that the hexatriene quantum yield is reduced to 30%, if the molecule is directly excited to the 2A state (and passes through the *C*_2_‐CoIn), fully consistent with the X‐ray diffraction experiment [131]. On the other hand, Hofmann and de Vivie–Riedle calculated earlier (with a reduced Hamiltonian, using only two coordinates) the hexatriene yield is higher, if the wave packet passes through the *C*_2_‐symmetric CoIn [162].

An alternative interpretation invokes a higher‐lying part of the IS, which part of the wave packets from the higher‐lying Rydberg states could reach: It was called CoIn_bu_ (“bu” for s‐cis butadiene) by Robb and coworkers [18] and previously kink‐CoIn or Hula‐twist Coin (Section 3). It was originally suggested by Tamura and coworkers [121]. As discussed in Section 5.2, it would not lead to ring opening but to internal conversion only, because it would be responsible for cis‐trans isomerization (Section 3), which is blocked in this case by ring strain immediately after passing this CoIn. The accessibility of an IS part corresponding to frustrated cis‐trans isomerization at higher excitation energies seems to provide a simpler interpretation of the increased internal conversion.

To summarize Section 5: The van der Lugt–Oosterhoff model with a two‐electron excited pericyclic minimum (2A), augmented with conical intersections, seems now established for electrocyclic reactions. The information on slopes and directions of motion in CHD ring opening came mainly from the observed coherent oscillations. A symmetry‐breaking distortion of the molecule (seeming ring contraction or in‐spiraling, with formation of a three‐electron three center bond, according to calculations) is initiated on surrounding the first (1B/2A) CoIn (Section 5.2). The corresponding low‐frequency ring vibration shows that the ring is weakened but not yet open in the 2A state, before passing through the last CoIn. The first change of direction occurs on leaving the FC region: Only after sufficient twist of the C=C bonds the WH interactions set in and (in the case of CHD) the CH_2_—CH_2_ bond can open (Section 5.4). The idea of a delayed choice is also supported by the observed products: The WH rules alone would allow for CHD conrotatory ring opening (to *Z*‐hexatriene) or disrotatory ring closure (to bicyclo‐ [2,2,0]‐hexene). In fact, products of the former are observed with *C*_2_ symmetric conformers or derivatives (where conrotatory distortion is FC active), and products of the latter, if starting with *C*_s_ symmetric precursors (with their disrotatory FC active coordinates) (Section 5.4.1). In conrotatory ring opening, the WH rules would also allow both, clockwise or anti‐clockwise torsion; in substituted derivatives, the products would be different. But the slopes in the FC region accelerate the wave packet toward the direction of predistortion, as if the molecule would prefer the path of least motion (Section 5.4.1).

The driving forces (slopes) initially point in the direction of FC active coordinates, then are governed by WH interactions. The cusp near the 1B/2A CoIn then drives the symmetry breaking of the molecule, and the lower cone of the 2A/1A (S_1_/S_0_) CoIn causes the branching toward product and internal conversion.

On excitation of a higher‐lying (Rydberg) state, the quantum yield of ring opening was found to decrease [131]. For interpretation, the authors pointed to the different direction, under which the molecule reaches the last CoIn. An alternative interpretation suggests that with the higher excitation energy, part of the molecules can reach a higher‐lying region of the IS, i.e., a higher‐lying CoIn that is expected to be unproductive (Section 5.5).

## 6 Charge Transfer: 4‐Dimethylamino‐Benzonitrile and Similar Molecules

### 6.1 Femtosecond Time‐Resolved Experiments

For the photochemical reactions of the preceding sections—on cis‐trans isomerization and pericyclic reactions—there were quantum chemical predictions of potentials and in particular of CoIns. They gave a very helpful orientation for interpretation of the time‐resolved experiments. Our measurements often confirmed them, but also sometimes caused further theoretical investigations. There was much less theoretical information on charge transfer (CT) reactions, before we began to investigate with femtosecond resolution the photochemical electron transfer in donor–acceptor substituted benzene derivatives such as 4‐dimthylamino‐benzonitrile (DMABN, formula in Figure 12 below) [163]. For CT reactions, no CoIn was known before. We could establish that after excitation to the S_2_ state (as is commonly done), the molecule relaxes through a CoIn (evidence: the short measured times) and branches there to two S_1_ minima (from where fluorescence was well known), one of them being the CT state. (The calculations done before, focused on other subjects than CoIns, in particular on equilibration between the two S_1_ minima). We also provided evidence for the involved coordinates, based on the observed coherent oscillations and by a resonance in the ion, vertically above the CT state [163, 164, 165]. Most of the coordinates were confirmed by the subsequent calculations. But some of them were also doubted and, more importantly, not all calculations are consistent with each other. Some more recent calculations are even inconsistent with our results (Section 6.2). It may therefore be helpful to remind of our evidence.

**FIGURE 12:**
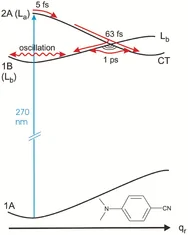
Suggested potentials and relaxation paths with the time constants measured by us [163, 164, 165]. A large component of the reaction coordinate *q*_r_ is the twist of the amino group, as discussed in the text. Taken from [165], with kind permission of RSC.

DMABN is a prototype of dual fluorescence and of photoinduced intramolecular charge transfer (see, e.g., the review of Grabowski, Rotkiewicz and Rettig [166]). One of the two fluorescing bands is only little Stokes‐shifted from the weak absorption to the S_1_ state (1B_2_ in *C*_2v_, L_b_ in Platt nomenclature, also “locally excited” or LE state). The other band shows a large Stokes shift; the corresponding upper state (second S_1_ minimum) has a large static dipole moment, consistent with an electron transfer from the donor (dimethylamino group) to the acceptor (benzonitrile group). The large dipole moment allows for energy tuning of this CT state by polar solvents. With suitable polarity, the two minima have comparable energy and can equilibrate. Hence the large Stokes shift must be due to a large displacement along a “reaction” coordinate, whose nature was controversial for a long time [166]. It is now mostly accepted that it involves (besides other contributions) a twist of the amino group relative to the ring (see also below); this view is also supported by all quantum chemical calculations.

Whereas the polarization of emission from L_b_ is perpendicular to the molecular axis (although due to Herzberg‐Teller coupling to the L_a_ state it contains a small parallel component), the CT emission is parallel‐polarized (see the Figures 30 and 31and context in the Grabowski review [166]). Hence, the CT state lies on the 2A_1_ surface. Also, the strong absorption to the S_2_ state (normally used to excite the molecule) is parallel polarized and leads to a state (L_a_), which lies on this surface.

Whereas most previous experiments investigated the L_b _→ CT reaction or equilibrium in solution, we monitored the process already from the initially excited (higher‐lying) L_a_ state, using time‐resolved mass spectroscopy with non‐resonant photoionization in the gas phase. We found [163] that the molecules leave the FC region in about 5 fs then reach a CoIn after another 63 fs, where the path branches to the L_b_ and CT states; subsequently the populations of the two states equilibrate within 1 ps (Figure 12).

The L_b_ state was identified by the two coherent oscillations observed there [164] (slightly updated in [165]), which were identified before by high‐resolution spectroscopy as amino group twist and pyramidalization in this state.

The signal from the location designated “CT” in Figure 12 showed an anisotropy with polarization parallel to the molecular axis (parallel to the pump laser). It must be due to a resonance with the probe laser (in this experiment 2100 nm, 0.59 eV); ionization without such a resonance is isotropic, and the signals from the other locations were in fact isotropic. This anisotropy rises within a time not far from 68 fs and decays again within about 1 ps, just as the population and depopulation times of the location labeled “CT” in Figure 12. Obviously, this location with its energy between L_a_ and L_b_ is strongly displaced from the others, and the only state in this energy range and strong displacement that is known is the CT state. Because the neutral molecule has no states lying so close together, the resonance must be ascribed to the ion; excitation of the ion leads to enhanced fragmentation. (In fact, the *molecular* ion shows no anisotropy).

The small energetic distance (0.59 eV) of the two ionic states (or of the corresponding orbitals of the neutral molecule) also allows for a conclusion on the reaction coordinate *q*_r_: distortion along it must bring two orbitals close enough together. This is the case for the orbital pair HOMO and HOMO–2, if *q*_r_ is the amino group twist: They are π‐antibonding and π‐bonding, respectively, between the amino group and the ring, and at a twist angle of 90° they would be degenerate, if no other deformation took place in parallel. The corresponding ionic transition is parallel‐polarized. We consider this to be strong evidence that the twist dominates in *q*_r_, because no other deformation could be figured out, which would bring another orbital so close to the HOMO (or another state of the ion so close to its ground state).

A strong support that *q*_r_ (or the path via the CoIn) contains the twist comes from the observation [164] that after arrival in the L_b_ state the wave packet oscillates in direction of the twist vibration. This coherent oscillation is excited by the slope from the CoIn downward. Hence, the molecule at the CoIn (or the location of passage through the IS) must also be twisted. (The L_b_ state is not twisted).

We also observed a coherent oscillation along the amino group inversion (pyramidalization) in L_b_ and concluded that the CoIn is also displaced in this direction [164]. Then the coupled‐cluster calculation of Hättig and Köhn predicted for the CoIn a pyramidalization of the ring carbon C_4_ carrying the amino group [167]. Obviously, this local deformation excites a normal vibration, involving the amino group inversion as well as a nonplanar arching of the whole molecule [165].

Besides DMABN, we also investigated derivatives (Scheme 6), in which the dimethylamino group is replaced by NH_2_ (ABN) or is integrated in a saturated ring (PIPBN, PYRBN), or where the ring is fused to benzene (NMC6). The results are compiled and discussed in [165].

**SCHEME 6:**
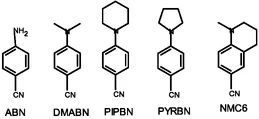
Some amino‐benzonitriles investigated in [165].

In all these molecules we observed the same reaction scheme, even with similar time constants: After leaving the FC region, the path branches at a CoIn toward L_b_ and CT; the CT state is populated only transiently and is then depopulated toward the L_b_ state. This is especially remarkable for ABN and NMC6: Their CT states lie higher than L_b_, because the NH_2_ group in ABN has only a small donator strength or because in NMC6 the twist causes steric strain. Population of the CT state via L_b_ would lead to an equilibrium with negligible CT population. By contrast, we observed a strong transient CT signal with the characteristic resonance and anisotropy.

In all these molecules, we also observed coherent oscillations, which must be assigned to vibrations in L_b_. Only this state has a lifetime, which is long enough to be compatible with the long damping time of the oscillations. The number of observed frequencies was different in the different molecules (for reasons discussed in [165]). Five frequencies were observed in ABN, two of them apparently overtones of others at lower frequencies. The three at lower wavenumbers agree well with published calculations for the L_b_ well, which also assigned them; two of them were also established (with assignment) by published fluorescence excitation spectra. One of the vibrations is of quinoid type with its a_1_‐symmetric changes of bond lengths in the ring and to the substituents; it is expected to be FC active. Another is a b_1_‐arching of the molecule; it involves amino‐group inversion in other molecules, in which this group carries substituents heavier than hydrogen. The third is the Kékulé vibration (b_2_ symmetry), in which the ring CC bond lengths change their lengths alternately. An amino group twist was not observed in this molecule; obviously its frequency would be too high to be time‐resolved, in contrast to the molecules with heavier amino groups, where it was observed.

In [165], we said that these vibrations are stimulated by slopes on entering L_b_ from the CoIn (or earlier in the case of the FC active quinoid vibration), a view that is supported by the calculations of Köhn and Hättig [168]. This view is slightly modified here: “crossing region of the IS” is used instead of “CoIn.”

Obviously, the corresponding vibrational coordinates must be components of the vector pointing from L_b_ to the crossing region of the IS. They must also be components of the branching plane vectors. We suggested that the Kékulé coordinate (b_2_ in *C*_2v_) is contained in the non‐adiabatic coupling (NADC) vector (as already suggested by Zilberg and Haas [169]) and the others (quinoid, arching, twist) in the gradient difference (GD) vector. The twist *must* be part of the GD vector, because the CT state is strongly displaced from L_b_ along the twist (90° according to many calculations, consistent with the long wavelength ionic resonance found by us), and the CoIn is between L_b_ and CT, and branching at the CoIn leads to the non‐twisted L_b_ state and the highly twisted CT state. By symmetry, a combination of twist (a_2_) and arching (b_1_) could also be part of the NADC vector (b_2 _= a_2 _× b_1_).

The branching plane is two‐dimensional, hence spanned by only two linearly independent vectors. Each vector may, however, be a fixed composition of others (examples at the end of this section) so that all the coordinates found above could be contained in the branching space. On the other hand, the trajectories may hit the IS at different crossing points, whose branching planes may differ. Then one single path might not excite all vibrations, but they would be excited by all paths together. However, it is hard to imagine that superposition of several different paths would maintain the coherence observed in the oscillations.

Some arching may occur also in the CT state according to Köhn and Hättig [167]: they found that the state with 90° twist (species B_2_ in molecular symmetry *C*_2v_) can actually be slightly stabilized by pyramidalization of C_4_ (carrying the amino group). A similar distortion was found by the Robb group [19]. With or without such a deformation, the emission from the CT state would be perpendicularly polarized. To explain the observed parallel polarization (Figures 30 and 31 in [166]), one must postulate (in addition) a pyramidalization of the amino group, even if this may take place only by thermal energy. In fact, Sudholt, Sobolewski and Domcke found by CASSCF/CASPT2 calculations that a CT state with 90° twist is further stabilized by such a pyramidalization [170].

So, from coherent oscillations and other spectroscopic observations (anisotropy, ionic resonance) we inferred quite a number of geometric details, both of the CT state and the CoIn or an IS range (and the slopes from there). By time‐resolved X‐ray diffraction of 4‐di‐isopropylamino‐benzonitrile (which shows dual fluorescence also in the solid state) Techert and Zachariasse found that there is practically no twist in the CT state [171]. On the other hand, Zinth, Elsaesser and coworkers found that such experiments suffer from an intermolecular effect: a strong dipole moment, optically induced in one molecule, strongly interacts with neighbor molecules [172]. Hence, a gas‐phase measurement would be desirable.

It should be mentioned that in two other experiments a direct population of the CT state by relaxation from L_a_ was seen, in our interpretation via branching at a CoIn: Kwok and coworkers found that the CT state of DMABN in benzene was transiently populated simultaneously with L_b_ [173], and Park, Kim and Joo saw simultaneous CT and L_b_ formation for DMABN in acetonitrile by time‐resolved fluorescence [174]; but the idea of branching at a CoIn was not mentioned. To interpret the latter measurements, the authors also invoke besides the fully twisted CT state a partially twisted one; perhaps they want to check, whether it can be interpreted as a hot CT state (cooled within 4.8 ps) so that the 430 nm emission would correspond to hot bands. For reaching the L_b_ and CT states, their ≤30 fs in acetonitrile seem fully consistent with our 68 fs, because in their solvent, the CT state is lower, the slopes are hence steeper, and the CoIn is therefore met earlier.

To summarize, our ionic resonance accessible from the CT state tells that the CT state is highly twisted, perhaps by 90°. In addition, the polarization of the CT emission requires that the amino group is pyramidalized, perhaps only with some thermal excitation. The fact that in the CoIn the path branches to the highly twisted CT state and the nontwisted L_b_ state means that the branching space (at this point of the IS) contains the twist. The observed oscillations mean that the CoIn (the region of the IS, preferred by the wave packet) is displaced along the twist, the arching and the quinoidal coordinates (probably all three are also components of the gradient difference (GD) vector, with weights varying in the IS); the Kékulé coordinate was suggested to be a NADC vector component of the branching space. Because GD and NADC vectors are not necessarily orthogonal to each other, some of the former coordinates might also contribute to the NADC vector. In fact, Gómez and coworkers [19] show an NADC vector, which is a superposition of the Kékulé and quinoidal coordinates. A similar superposition was calculated by Georgieva and coworkers for this vector, and a superposition of quinoid and an asymmetric planar ring deformation for the GD vector [175]. An investigation by time‐resolved diffraction in the gas phase would be desirable to check the transient deformations.

### 6.2 Quantum Chemical Calculations

As already said, all calculations say that in the CT state of DMABN the amino group is twisted by 90°, consistent with our results. The Robb group finds in addition a nonplanar ring distortion in this state: a pyramidalization of C_4_ [19]. Also Köhn and Hättig [167] found a similar additional distortion in the CT state, whereas Sudholt, Sobolewski, and Domcke find an additional slight stabilization by amino‐group pyramidalization [170], which can explain the parallel polarization of CT fluorescence (Section 6.1).

There is also agreement with our results that a CT state exists—and is accessible from L_a_ via a CoIn—also in ABN [19, 176] and NMC6 [168].

There is much less agreement on the location of the L_a_‐CT/L_b_ CoIn and the slopes nearby:

1. One group of calculations [19, 167, 175] says the CoIn (MECI) is displaced from the L_b_ minimum by about 45° along the twist and by additional distortions (quinoidal bond length changes, pyramidalization of C_4_ [167] or C_3_ and C_5_ [19]).

As already discussed in [165], the calculated CoIn displacements (toward the GD direction) and our observed oscillations support each other, because the vibrations are excited by the slope from the CoIn (the Kékulé vibration by the slope in NADC direction). Note also that not all calculations looked for additional distortions.

2. Gómez, Robb and coworkers [19] investigated a larger part of the IS by CASSCF combined with RASSCF (which takes more dynamic correlation into account). They found that the S_2 _→ S_1_ relaxation does not necessarily need the twist: he IS can be reached in a large range of twist angles (the MEP in the IS is nearly parallel to the MEP on S_2_), the MECI displacement does not contain the twist, and this coordinate was also not contained in the branching space. (But the other deformations mentioned above were found, too). Nevertheless, the CT state was found to be 90° twisted. They also suggested that the twisting begins early in S_2_ (referring to Köhn and Hättig [167]) so that the wave packet crosses the IS in a twisted conformation.

In a later work on the CASCF/CASPT2 level [176] in the gas phase and in acetonitrile, Gómez and coworkers point again to the extended IS, but suppose that the molecule prefers an S_2_/S_1_ internal conversion without twist, that is a path L_a _→ L_b_ and only then L_b _→ CT. This path is contested in comment (3) below.

We point out that (a) the observed twist oscillation in L_b_ requires a corresponding slope from a CoIn; hence it is twisted; and (b) the observed simultaneous formation of the nontwisted L_b_ state and the highly twisted CT state implies a CoIn with a branching space containing the twist as a component. It is worth noting that the calculations search for the energetically steepest slopes. In this respect, the twist is certainly less effective than any bond‐length changes so that it may elude the search. On the other hand, bond‐length changes may be reversed or elude the attention in the “product” states L_b_ and CT.

Georgieva and coworkers, using ADC(2) combined with MRCI, found a barrierless path from L_a_ to CT through a CoIn (MECI), which on the one hand is twisted, whose branching space on the other hand does not contain the twist [175]. The comment (b) above also applies to their results. Note that this work also presents a comprehensive discussion of DMABN calculations up to 2015.

3. In three more recent calculations, based on TDDFT or ADC(2) (which both place the CT state too low) and with calculation of trajectories and associated times, DMABN relaxes through a CoIn without twist from S_2_ (L_a_) to L_b_ and only then from L_b_ to CT [177, 178, 179]. (The work by Du and Lan, primarily devoted to software development, also finds internal conversion without twist [180]). Because their results are possibly the current view, it seems justified to comment on them. The arguments are based on evidence of Section 6.1.
  a. All three works say that we only assumed a twist as an active coordinate in and near the CoIn. We did not assume it but presented solid evidence for it. Section 6.1 compiles it again.
  b. We showed clear evidence that the L_b_ and CT states are populated simultaneously (not consecutively as in the L_a _→ L_b _→ CT mechanism), with the path obviously branching at a CoIn. The L_b_ population was recognized in DMABN by the characteristic frequencies of twist and pyramidalization vibrations, previously assigned by high‐resolution spectroscopy, the CT population by the anisotropy rising and decaying with the same time constants as the CT population itself. We consider the simultaneous population found by Kwok and coworkers in benzene [173] and by Park and coworkers in acetonitrile [174] a confirmation, although they do not mention a CoIn.
  c. All three works calculated very short times (around 10 fs) for the S_2 _→ S_1_ (L_a _→ L_b_) relaxation and tentatively identified it with our 5 fs. (We assigned it to departure from the FC region, Figure 12). For our next time (63 fs, the passage through the CoIn in our mechanism, Figure 12), they suggested the adiabatic charge transfer (L_b _→ CT). However, this process takes about 100 times longer in solution and has never been seen in the gas phase (it is endothermic there).
  d. All three works would not be able to explain the origin of the oscillations (vibrations) observed by us in L_b_.
  e. They would not be able to explain an only temporary CT population in the gas phase, observed by us. In their mechanism L_a _→ L_b _→ CT, the last step would be monotonic. Actually, it is an equilibration. An experimental work by the Zachariasse group [181] also postulates an L_a _→ L_b _→ CT path (with a vertical L_a _→ L_b_ relaxation). The same objection applies to their work, as also (b), (d), and (f). (The Zachariasse group investigated over the years the L_b_/CT equilibration, comparing different compounds. They believed to see evidence for a non‐twisted CT state. See the literature quoted in [181]).
  f. They would not be able to explain, how the higher‐lying CT states of ABN or NMC6 could be (temporarily) populated. We estimated the *equilibrium* population in ABN at room temperature to 5 × 10^–5^ relative to L_b_, which would not be consistent with our strong temporary CT signal.

To summarize Section 6.2: Although the quantum chemical calculations all support a twist of 90° in the CT state of DNABN, there is much less agreement for the CoIn; in most calculations the twist is not part of the branching space; instead, they say the degeneracy is maintained in this direction, in contrast to our experimental evidence. Perhaps it could help to investigate the model of the next section by calculations.

### 6.3 Why Is There Any Twist?

As just said, all the quantum chemical calculations support a 90° twist for the emitting CT state. But they did not ask or answer the question, why should there be any twist? In fact, it is counter‐intuitive at first glance: After taking away one electron from the amino group, the resulting positive nitrogen is tetravalent and can form a double bond to C_4_, and this double bond would prevent a twist (as indicated in the following Figure 13).

**FIGURE 13:**
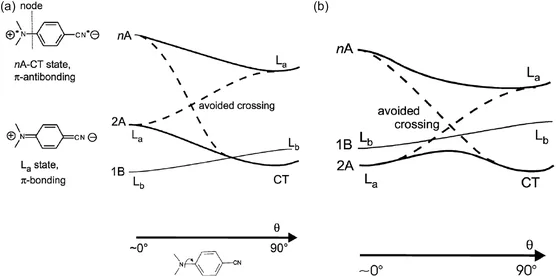
A model postulating a π antibonding CT state (*n*A) above the π bonding L_a_ state. (a) If along the twist, the crossing is avoided strongly enough, it generates a down‐slope also for the 2A (L_a_‐CT) potential. (b) In polycyclic analogs, L_a_ is below L_b_, and the crossing is more weakly avoided so that an activation barrier arises on the L_a_‐CT path. Both diagrams were taken from [165], with kind permission of RSC.

We suggested [165] that above L_a_, there is a higher CT state of the same symmetry (*n*A_1_), which is π‐antibonding between N and C_4_. (We argued, a molecule‐in‐molecule consideration predicts such a state). Twisting would lower its energy. If the upper and lower states strongly interact vibronically and thus repel each other, the lower (L_a_‐CT) potential will also go down along the twist direction. Because the suggestion has not found much attention, the figure from [165] is reproduced here (left side of Figure 13).

We argued that there are not only some observations indicating such state mixing but that also quantum chemical calculations [182, 183] support the idea. (See [165] for details). In the meantime, there is another calculation, which includes higher states as a function of twist [184]. Their potentials resemble those in Figure 13. But none of the calculations considered explicitly any vibronic interaction of upper and lower states (or the parentage of the resulting states). For further insight, it would also be desirable to transform molecular wave functions into valence‐bond structures such as done by Robb and coworkers [19].

The model predicts an early driving force not only along the twist but also along the other coordinates discussed in Section 6.1 [165]. Furthermore, one can apply it to donor–acceptor systems, in which the benzene ring is replaced by a polycyclic aromatic. In them, the L_a_ state is lowered to below the L_b_ state and, due to delocalization, the repulsion between upper and lower potentials is weaker; in this case the model predicts not only a CT state with perpendicular twist (TICT) but also another one which is planar (called MICT, for mesomeric intramolecular charge transfer [166]), and an activation barrier between them due to the more weakly avoided crossing [165] (right‐hand side of Figure 13). Such MICT states including the barrier have been observed. The model rationalizes them [165].

Hence, the model for the first time suggests an origin for a driving force toward a twist.

To summarize Section 6: We have solid evidence not only that the CT state is twisted (from the observed anisotropy and the resonance) but also that the twist plays a crucial role for the branching at a CoIn or IS region, leading to simultaneous population of the twisted CT state and the non‐twisted L_b_ state. From the observed oscillations in L_b_, we infer slopes from the CoIn not only toward twist but also toward quinoidization (FC active), arching (including amino‐group pyramidalization) and the Kékulé vibration (component of the NADC vector). The twisted CT state (with possible additional distortions) agrees with all calculations. For the displacement of the CoIn, except the twist all coordinates found by us occur in the calculations with varying weights. We suggested to consider vibronic coupling with a higher CT state to explain an early driving force also toward twist. More recent calculations, suggesting a very fast L_a _→ L_b_ relaxation and subsequent L_b _⟷ CT equilibration are not compatible with our results.

## 7 Metal Carbonyls

Before we began studying the ultrafast dissociation of metal carbonyls, structures of CoIns had not been calculated. It was, however, easy to guess some of them: Caused by the high symmetry of many of the involved species, there are many degenerate states, which are subject to Jahn–Teller splitting, with the active coordinates suggested by group theory. These are just CoIns. Additional intersections can be expected due to the large number of close‐lying states in the UV region. Many details that we found for the reaction path are in the meantime confirmed by calculations and by time‐resolved diffraction methods or other advanced techniques. McKinlay and Paterson reviewed computations on binary metal carbonyls with emphasis on the role of the Jahn–Teller effect, considering also ultrafast photoprocesses [185].

Recently the ultrafast photochemistry of binary metal carbonyls was reviewed by Ramasesha and coworkers [186].

We investigated the dissociation of M(CO)_6_ (M = Cr, Mo, W) [187, 188], Fe(CO)_5_ [189], Ni(CO)_4_ [190], and M_2_(CO)_10_ (M = Mn, Re) (brief survey in [191]) in the gas phase with excitation mainly at 267 nm and probing by 800 nm multiphoton ionization and mass selective detection. The time resolution in this initial work was around 100 fs. (As explained in Section 2.2, we could nevertheless extract time constants in the 10 fs range but not high‐frequency oscillations). Later, we reinvestigated M(CO)_6_ with 10 fs resolution, Cr(CO)_6_ also with a number of different pump wavelengths [22]; the shorter pulses not only confirmed the time constants but also revealed higher‐frequency oscillations. Our work was also reported in the review by Long [192], more briefly by Turner and coworkers [193], and the Fe(CO)_5_ work (with comparison to diffraction experiments and calculations) by Ischenko and coworkers [12].

The UV pump populates one of the metal‐to‐ligand charge transfer (MLCT) states, in which a d electron is excited to a π* (metal–C bonding) orbital. For dissociation, this population must flow to one of the (optically more or less dark) repulsive ligand‐field (LF, d → d or metal centered, MC) states, in which the higher orbital is σ antibonding. In Cr(CO)_6_ the LF states lie above most MLCT states but are expected to fall steeply down on Cr–C extension (see [22]; a more recent calculation [194] predicts LF states also among the MLCT states). In Fe(CO)_5_, the lowest excited state has LF character [195].

To show an example, Figure 14 presents the potentials and the dynamics for Fe(CO)_5_ photodissociation, as suggested in [120]. The two alternative paths—initial relaxation down to a repulsive LF state or an early diabatic transition to a dissociative state—are represented by solid and broken lines, respectively [120]. The following discussion focuses on the case of iron carbonyl.

**FIGURE 14:**
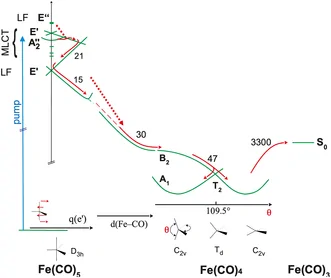
Potentials and dynamics in Fe(CO)_5_ photodissociation, as suggested in [120]. Traveling times are in fs. q and θ indicate pseudorotation coordinates before and after dissociation. The path indicated by solid lines was preferred in [120], because it seemed more compatible with the calculations of Banerjee [196]; the alternative path for the early part (broken lines) is favored in the electron diffraction work [197].

The following discusses some of our results and conclusions in view of more recent results and adds some more interpretations, in particular the question of triplet channels:

1. Only one CO is cleaved off photochemically, typically in a time of ≤50 fs (Figure 14). If there is enough excess energy in the remaining unsaturated carbonyl M(CO)_*n*–1_ (which includes all carbonyls investigated by us, also the binuclear ones), a second CO can be eliminated in the picosecond range (3.3 ps in the example of Figure 14) in the hot ground state. This second step is suppressed in the condensed phase. (It was long known that only one CO is eliminated in solution [198], a fact which we used for assignment). Indeed, exciting Fe(CO)_5_ to its lowest state (which leaves nearly no excess energy), Ihee and coworkers found by time‐resolved electron diffraction only Fe(CO)_4_ up to more than 200 ps [199]. In the same system, with 267 nm excitation, Cole‐Filipiak and coworkers confirmed the stepwise dissociation (with similar time constants and wavelengths as in Figure 14) by time‐resolved IR spectroscopy in the gas phase [200], and similarly Leitner, Wernet and coworkers by time‐resolved X‐ray photoelectron spectroscopy [201, 202]. The stepwise process was also confirmed by ultrafast X‐ray diffraction [203].
2. The unsaturated carbonyl M(CO)_*n*–1_ is initially formed in its first excited singlet state S_1_. Whereas Ni(CO)_3_ (which has no easily accessible S_1_/S_0_ CoIn [190]) shows a beautiful red luminescence (phosphorescence, according to the lifetime >10 µs [190, 204]), those with higher coordination number (4 or 5) relax from S_1_ through a Jahn–Teller induced CoIn down to S_0_ within about 50 fs, following a pseudorotation coordinate (Figure 14). This assignment was based on the coherent oscillation observed with coordination number 5; its wavenumber coincided with that of the known pseudorotation vibration in Cr(CO)_5_ in its S_0_ state. This path explains some phenomena in photosubstitution such as the cis effect, which puzzled many workers before (discussed in [120, 188]). The path from S_1_ to S_0_ in Cr(CO)_5_ including the pseudorotation oscillation was confirmed by the calculations of Paterson and coworkers [205, 206]. In the unsaturated Fe(CO)_4_ we did not see any oscillations; we supposed the S_1_–S_0_ distance too far from resonance with the probe laser But Paterson and coworkers calculated in the meantime that there would be a suitable resonance [195]; it is very broad, however, so that the probe transition would not be modulated by vibration. But we assumed the same scheme as in the group‐6 carbonyls, because the number of steps was the same and the time constants and the accompanying change of fragmentation patterns were very similar. In fact, in a recent ultrafast X‐ray scattering a coherent pseudorotation oscillation (78 cm^–1^) was also seen in Fe(CO)_4_ [203] (see also the discussion in [120]).
3. All processes in the investigated times up to 500 ps take place in the singlet manifold (in the gas phase). If intersystem crossing played a role, one would expect that it would be faster with heavier metals. But we observed the opposite with the carbonyls of Cr, Mo, W and with those of Mn and Re. For the chromium system we also argued with the frequencies of the observed pseudorotation vibration, which coincided with that known for the singlet Cr(CO)_5_. The ground state of this unsaturated carbonyl is a singlet. The singlet pathway in this system has apparently been accepted.

In the binuclear carbonyls M_2_(CO)_10_, M = Mn or Re one observes [198] metal–metal bond splitting besides CO elimination, with wavelength dependent quantum yields: CO elimination is favored at shorter wavelengths. The homolysis would require a triplet of the binuclear carbonyl, if both fragments M(CO)_5_ were produced in the electronic ground state. This is what was assumed in the literature [198], also by the Zewail group in ultrafast spectroscopy of Mn_2_(CO)_10_ [207]. Whereas we did not identify signals indicating homolysis at our pump wavelength of 267 nm, the Zewail group probably saw one, pumping at 310 nm, with time constant below100 fs [207]. This time is too short for intersystem crossing. Instead, it is suggestive [191] to assume that one of the M(CO)_5_ is produced in the first excited state (a process which would be allowed in the singlet manifold) and then relaxes down again via a pseudorotation CoIn, just as also the M_2_(CO)_9_ fragment produced in competition [191].

The wavelength‐dependent quantum yields for the two channels probably deserve new attention of quantum chemists: It obviously involves an early branching. Either two different electronic states (but no triplet) can be excited, or two different momentum directions are possible in one state. In organic photochemistry, an example of clearly different electronic states is the site‐selective dissociation of a di‐iodide [208], whereas many of (the more frequent) momentum effects in the FC region are compiled in [17]; see also the wavelength dependent photochemistry of alkenes in Section 4.3.

In contrast to the corresponding chromium carbonyl, the ground state of Fe(CO)_4_ is a triplet (T_1_; the numbering T_1_ and S_0_ is preferred, because they correlate with T_1_ and S_0_ of Fe(CO)_5_); the T_1_–S_0_ distance is not experimentally known but probably small (near 0.1 eV [209]). We argued that Ryther and Weitz in gas‐phase transient IR spectroscopy after many nanoseconds detected both, triplet and singlet Fe(CO)_4_ (distinguished by their very different rate of CO addition) [210, 211]; if intersystem crossing took place in the picosecond or sub‐ps range, no singlet would be left over after many nanoseconds.

Our claim of a pure singlet path in the gas phase (up to at least 500 ps) has triggered many investigations, also because some sub‐picosecond times for intersystem crossing were known in the condensed phase, and more such short times are being detected.

3a. When Ihee, Cao and Zewail with their (mentioned) electron diffraction had improved the time resolution enough to detect the primary photodissociation product of Fe(CO)_5_, they found a structure that did not agree with that known for the ground‐state (triplet) Fe(CO)_4_; in particular, the bond angles were different. Encouraged by our claim of a singlet path, they then compared their data with a structure calculated for singlet Fe(CO)_4_ and found very good agreement [199]. This is a good example, how time‐resolved spectroscopy, time resolved diffraction and quantum chemistry can supplement and support each other. It is also remarkable that in this case a diffraction method was able to recognize the electronic state.

As said, Ihee and coworkers found no change of their structure over the investigated time of 200 ps. This is evidence against intersystem crossing in the gas phase in this time span.

In their highlight, Poliakoff and Turner [212] tell the story, how the ideas on the structure of Fe(CO)_4_ developed over time.

3b. The mentioned work using X‐ray photoelectron spectroscopy [201, 202] not only confirmed the stepwise CO extrusion but also that it takes place in the singlet manifold in gas‐phase photodissociation of Fe(CO)_5_ near 266 nm. (See also the simulations of these spectra in [213]). The same conclusion was drawn from gas‐phase transient IR spectroscopy [200], although in this case a time constant of 15 ps was tentatively assigned to intersystem crossing.
3c. On the other hand, Troß and coworkers in their time‐resolved X‐ray absorption experiments find signatures (in their Figure 7) that also the triplet ground states of Fe(CO)_4_ and Fe(CO)_3_ contribute to their spectra [214]. In accompanying calculations they point to a crossing of the singlet and triplet potentials of Fe(CO)_4_ along an angle deformation coordinate, where intersystem crossing might take place. The authors conclude that a fundamental reexamination of the system would be worthwhile.

In the ultrafast X‐ray diffraction, Schori and coworkers [203] also found coherent bending oscillations (main component 78 cm^–1^) after dissociation; the spectrum would be consistent with a minor (13%) contribution by triplet Fe(CO)_4_, according to calculations by the authors. But recently, it was suggested [120] that, by analogy with the unsaturated Cr(CO)_5_, these bending vibrations are due to pseudorotation, stimulated by S_1 _→ S_0_ relaxation through the Jahn–Teller induced CoIn, and that additional frequencies belong to overtones and combination vibrations, all in S_0_ of Fe(CO)_4_.

To comment further on the suggested [214] intersystem crossing (ISC): If around 1 ps already 13% (=1 – exp(–0.14)) of the molecules had undergone ISC, after 200 ps (time window of Ihee and coworkers [199] in which they only detected a singlet) no singlet (namely exp(–0.14·200)) would be left over .

3d. In condensed phase, ISC seems to be much faster. Many papers investigated photosubstitution of Fe(CO)_5_, i.e., ligand addition to unsaturated carbonyls (see the Introduction in [215]). In transient IR studies of the Harris group with 0.3 ps resolution both, singlet and triplet Fe(CO)_4_ (recognized by their reactions) were found [216, 217], and both were assumed to be primary products. Also the George group investigated this system in the solvents heptane, supercritical xenon (scXe) and scAr by picosecond and nanosecond time‐resolved IR spectroscopy [218]. They found triplet Fe(CO)_4_ and Fe(CO)_3_ are formed before a few picoseconds. (The Fe(CO)_3_ was interpreted by dissociation of the hot tetracarbonyl, which seems possible in solvents with very long cooling times). But they also saw minor quantities of products that are apparently formed from primary singlet Fe(CO)_4_. In a later work [215], the same group reinvestigated this system in more detail, determining not only rate constants for the ligand addition reactions but also activation energies (together with DFT calculations). They still assume that *triplet* Fe(CO)_4_ is the (main) primary product. There seems to be a conflict between its rapid formation in condensed phase (in about 300 fs) and its much slower formation in the gas phase (>200 or 500 ps).

But in the same work [215], they present a scheme, which should solve the conflict: As a function of distance to the solvent or new ligand, the potential of the singlet is purely attractive and barrierless, whereas the (slightly lower‐lying) triplet is purely repulsive. At the crossing point of the two curves, intersystem crossing is expected to be particularly fast. Ligand (L) addition to triplet Fe(CO)_4_ will first have to climb up to this point (which explains the activation energy) to form singlet Fe(CO)_4_·L, whereas it forms from the singlet directly without barrier (Figure 15 bottom right).

**FIGURE 15:**
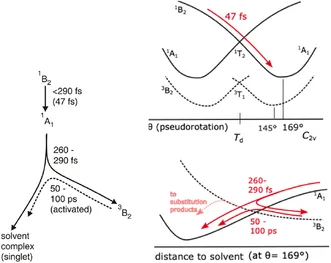
*Left*: A branched path for forming with the same time constant both, a solvent complex of singlet Fe(CO)_4_ and the uncomplexed triplet of this species. The process is induced by collisions with the solvent. *Bottom right*: The same process is shown, now as a function of solvent–Fe distance. It shows that the path from the singlet is barrierless, whereas from the triplet, complex formation needs activation energy. *Top right*: The potentials (versus the pseudorotation coordinate) and path for the preceding formation of Fe(CO)_4_ in its S_0_ (^1^A_1_) state, in the gas phase (Figure 14), here suggested to apply also for the condensed phase.

This offers a solution of the conflict (left hand side in Figure 15): Suppose also in solution the only dissociation product is the singlet, formed in a time ≤100 fs (<290 fs in the figure). When on approach of the solvent it arrives at the crossing point, the path will branch and form with the same time constant (ca. 300 fs) singlet Fe(CO)_4_·L and uncomplexed triplet Fe(CO)_4_.

That is, the approaching solvent brings the singlet species to the crossing point of singlet and triplet and thus brings about the sub‐ps intersystem crossing, without additional heavy‐atom effect. It seems to me that this model is consistent not only with the investigations of the George and Harris groups above. It would also explain the following results of the Wernet group found by time‐resolved X‐ray spectroscopy:

In ethanol solution they found that the same time constant (290 fs) for the tetracarbonyl spin cross‐over (to ^3^B_2_ Fe(CO)_4_) as for ligand association (to form singlet Fe(CO)_4_·HOC_2_H_5_) [219, 220, 221]. An identical time constant is strong evidence for a branched path with one common precursor, such as that suggested in Figure 15. The authors instead suggested formation of ^3^B_2_ directly from the naked singlet Fe(CO)_4_. But then it should occur in the gas phase with the same time constant, which is inconsistent with the gas‐phase experiments discussed above. The proposed mechanism is in harmony with transition‐state theory for spin‐forbidden processes (which, however, does not consider the paths coming from higher energies in Figure 15). Harvey [222] compile applications of this theory to a number of examples, which include the addition of CO to triplet Fe(CO)_4_ to form singlet Fe(CO)_5_.

Even for a barrierless process, the association of the new ligand with singlet Fe(CO)_4_ is remarkably fast, clearly faster than collisional cooling, which takes a few picoseconds. This is discussed in [221]. The authors compare it with an estimated collision time (500 fs) and ask, what brings the two molecules into correct orientation. This question is perhaps answered by the finding of the Rose‐Petruck group (by infrared and X‐ray techniques) that alcohols and other potential ligands form a weak complex with iron pentacarbonyl already before dissociation (see, e.g., [223, 224, 225]), which will help not only with the orientation but also with a proximity effect.

4. As said above, UV excitation initially populates an MLCT state, because such a state of suitable symmetry carries a high transition moment. Whereas such states are bound, LF states are dissociative but are optically nearly dark. We suggested, due to the density of states and due to Jahn–Teller splitting, there will be no problem for the molecules to find a suitable intersection (CoIn), so that a fast (one‐ or two‐step) internal conversion MLCT → LF can take place before dissociation.

In anti‐Stokes X‐ray Raman scattering with very high time resolution Kunnus and coworkers in fact could detect in Fe(CO)_5_ an initially populated MLCT state followed by a dissociative LF state [221].

With similar high time resolution (58 fs), Schori and coworkers investigated Fe(CO)_5_ photodissociation by ultrafast X‐ray diffraction [203]. The results are admirably detailed: They not only saw all the different steps—from the predissociative part (passing from MLCT to LF) via the primary product (singlet) Fe(CO)_4_ with its dynamics (including vibrations, some assigned [120] to pseudorotation) to the secondary CO elimination—but also energy partitioning to translation, rotation, and vibration of the dissociating CO. They saw dominating expulsion of an axial CO. Remarkably, they also find stretch oscillations of the Fe—C bonds (“predissociation oscillations”), followed by synchronized prompt ejection of a CO molecule, which rotates. The observed anisotropy indicates extrusion of an axial CO. Our pre‐dissociation times (15 + 21 fs, Figure 14) may be compared with their *τ*_0_ = 43 fs and our subsequent time (30 + 47 fs) to reach S_0_ of Fe(CO)_4_ with their *τ*_1_ = 94 fs.

An electron diffraction study of the same system [197], with less time resolution (150 fs), focused on the equatorial dissociation (following the axial channel), whose delay was clearly demonstrated by the decay of anisotropy (dissociating bond direction compared with pump laser polarization). This channel must be preceded by pseudorotation in the parent molecule, as pointed out by the authors [197].

In a preceding semiclassical dynamics calculation [196] the periodic bursts of CO ejection, synchronized with the bond length maxima of an Fe–C stretch vibration, were predicted and appeared even more clearly. The bond stretching triggers the nonadiabatic MLCT → LF transitions, and the authors speak about a ballistic motion. The majority path leads to extrusion of an axial CO. (In the TDDFT calculation, a – bound – A_2_” state is excited; the repulsive LF E’ state, reached thereafter, involves an axially antibonding d orbital). The minority path with its equatorial dissociation is activated by pseudorotation, which is a Jahn–Teller (JT) active coordinate for the E’ state. The ejected CO rotates. That is, already before repulsion begins, the Fe–C–O group is bent, which must also be caused by JT distortion in the rapidly populated E’ state [196].

The bond cleavage, synchronized with bond stretch oscillations, were suggested [120] to be more general (e.g., also in ring opening of cyclohexadiene) and ascribed to localized passage of a compact wave packet through or around CoIns; extensive use was made of slopes near CoIns, often caused by the JT effect. For example, the coherent oscillations (slightly reassigned) in S_0_ of the dissociation product Fe(CO)_4_ was ascribed to pseudorotation, stimulated by the slopes at the JT induced S_1_/S_0_ CoIn, similar as in photodissociation of Cr(CO)_6_ and other metal carbonyls. The delay of the minority channel—elimination of an equatorial CO—was interpreted by the slowness of pseudorotion in the parent molecule. The early acceleration in this direction (inferred from the rotation of the extruded CO) was associated with the NADC vector at the crossing of the excited A_2_” potential with a close‐lying MLCT E’ state or with the rapidly falling LF E” potential (solid and broken lines in Figure 12, respectively) [120]. Hence, this interpretation ascribes excitation of pseudoration to electronic forces, whereas Ma and coworkers [197] invoke general vibrational energy redistribution by anharmonicity; such a process, however, would destroy coherence that was observed over much longer times.

This path in Fe(CO)_5_ describes an S_*n *_→ S_1_ (A_2_” → E’) relaxation that is clearly not vertical: The rotation of the expelled CO requires an intermediate deflection. A vertical path was indicated in the calculation of Banerjee and coworkers [196] (in their Figure 1), probably only for simplicity.

In analogy to Cr(CO)_6_, a one‐step MLCT → LF relaxation (to the broken line in Figure 14) was also considered [120], although a two‐step process was preferred, because it is more compatible with the calculation [196]. In contrast to this path, on the direct way from the initial state to the broken line, there would be an MLCT/LF intersection along an Fe–C stretch coordinate so that triggering of dissociation by the stretch oscillation seems very intuitive. Such a direct path was favored in the electron‐diffraction work [197].

It is also remarkable that the proper dissociation step takes only 30 fs according to Figure 14. This is only a fraction of the Fe–C vibrational period (80 fs). This is another justification to call the dissociation ballistic [196, 203].

To summarize: The chapter on metal carbonyls is a good example, how ultrafast spectroscopy with various detection methods, quantum chemistry and advanced time‐resolved detection methods supplement each other. Help came also from end product analysis (only one CO is split off in condensed phase, hot reactions are suppressed though not completely) or from lower time resolution (triplet only in limited quantity after nanoseconds). Coherent oscillations again gave indications of directions of slopes and the reaction path. X‐ray techniques could investigate also reactions in the liquid phase. They confirmed that intersystem crossing can be much faster there (300 fs) than in the gas phase (>500 ps). It is suggested that the conflict is solved by realizing that an approaching potential ligand lifts the triplet surface but lowers the singlet surface; intersystem crossing occurs at the crossing point, without additional heavy‐atom effect. Great progress in time‐resolved diffraction methods has enabled very detailed insight into the motions of the atoms (including coherent oscillation, rotation of the expelled CO, pseudorotation, identification which CO is eliminated), energy partitioning (electronic, vibration, rotation, translation), and has even allowed to identify states (singlet versus triplet). Modern time‐resolved X‐ray spectroscopy could distinguish MLCT and LF states. Further progress can be expected, perhaps also to find out, how the known cases of wavelength dependent photochemistry work (e.g. homolysis versus CO elimination in the binuclear carbonyls).

## 8 Concluding Considerations

If the geometry of a CoIn plays such a crucial role for directing the molecule to the end products, it is suggestive to use their structure to examine calculated CoIn geometries. Several examples were given above for this idea:

• The conformers formed in photoisomerization of conjugated polyenes support the predicted Hula‐twist CoIns (Section 3). Sometimes, calculations find a CoIn involving a pyramidalization of one carbon in addition to double‐bond twist (e.g., in stilbene) and are believed to support a one‐bond flip. However, also Hula twist with its approach of C_1_–C_3_ involves a strong pyramidalization of the enclosed C_2_. It was also reminded in Section 3 that the reaction is only half finished in the CoIn; the molecule can return from there to the reactant, but also turn to one‐bond flip or to rotamerization.
• From the formation of carbene products in parallel to cis‐trans isomerization, it was concluded that in the CoIn (or IS) for alkenes and ethylene both, a double‐bond twist and a partial 1,2‐migration of H or an alkyl group play a role (Section 4).
• The end products also allow for conclusions on other parts of the pathway, such as the separation of FC induced forces (slopes) from those of reactive forces, e.g., from Woodward‐Hoffmann interactions. This principle allowed to explain, for example, when the molecule prefers clockwise versus counter‐clockwise rotation of double bonds, or conrotatory ring opening versus disrotatory ring closure (Section 5).
• From the observed strong twist of the amino group in the charge transfer state (kind of primary product) of DMABN‐type molecules, a twist was also inferred for the CoIn, in contradiction to many calculations but supported by our experimental findings (Section 6.1). A model (Section 6.3) suggests an origin of the force driving the twist (vibronic interaction with a higher, π‐antibonding state).

The displacement of a CoIn can also be inferred from coherent oscillations, if they are stimulated by the slope on leaving the CoIn. Thus, the observed oscillations in DMABN‐type molecules indicated that the CoIn involves a twist of the amino group and a number of other coordinates (Section 6.1). And the pseudorotation oscillations observed after dissociation of metal carbonyls confirmed that the primary product is S_1_ of the unsaturated carbonyl, which relaxes to S_0_ via a symmetry‐induced CoIn (with pseudorotation as symmetry‐breaking coordinate), which was confirmed by calculations (Section 7). Such a path is probably valid not only for binary metal carbonyls.

The idea of slopes or forces (a concept that is familiar to chemists) gives the chance to understand, what drives the wave packet in the observed direction, eventually to the observed product. Four such slopes were distinguished:

1. Slopes in the FC region. They are totally symmetric. They are often concluded from spectroscopy.
2. Forces associated with a reaction, such as those for pericyclic processes, for cis‐trans isomerization (be it Hula twist or not) or the repulsion responsible for dissociation. They are typically activated only after some FC deformation. (This is practically a generalization of the concept of predissociation). They often accelerate the molecule to a CoIn. After a first CoIn, they can again be active in a two‐electron excited state and bring the molecule to a second CoIn.
3. Slopes near a CoIn along the coordinates of the branching space. Note that the two vectors spanning the branching space can each contain several coordinates as components (examples in Section 6). An important case is, if the wave packet approaches the CoIn on the lower cone. Then, it goes around this cone with deflection toward the non‐adiabatic coupling vector. The idea was invoked at the first CoIn in ethylene (Section 4.1) and cyclohexadiene (Section 5.2), where it explained acceleration toward the second CoIn (or a region of the IS), breaking the symmetry of the molecule. It was also used to interpret the dependence on the initial state in alkenes (Section 4.3); a spectacular example are the cyclobutenes, where the stereospecificity of ring opening strongly depends on the initial state (Section 4.3.2, in detail in [17, 114]). (Note also that for this dependence, the deflecting forces are crucial; the presence of a CoIn alone is not a sufficient explanation, see Figure 6). In Fe(CO)_5_, the idea was used to explain a beginning pseudorotation and a bending of a Fe–C–O group (causing rotation of the expelled CO) before dissociation (Section 7). The slopes caused by the Jahn–Teller effect in a degenerate state are a special case of those near a CoIn (Section 7). Nenov and de Vivie‐Riedle extended the two‐electron two‐orbital model to rationalize and qualitatively predict structures at CoIns [226, 227]. Their method also seems promising for slopes near CoIns.
4. Forces by the σ backbone (not involved in excitation) or by the environment. They are often rather weak, sometimes causing a double minimum (two conformers). But the resulting small distortion can make the corresponding coordinate FC active, so that on excitation this distortion is enhanced. This principle of amplified predistortion explained many previous observations and rules that were only empirical before (Section 5.4.1; more details in [17]).

Of course, a driving force is a matter of interpretation. But quantum chemical calculations and time‐resolved diffraction methods also require interpretations. For example, if these two methods find that a certain cyclohexadiene derivative on excitation *opens* its ring to a *Z*‐hexatriene derivative (and deliver more details such as time constants or changes of atom distances), it is not yet explained, why the molecule does not *close* the ring to a bicyclic cyclobutene; some cyclohexadienes in fact do (Section 5.4.1).

Indeed, conventional ultrafast spectroscopy, time‐resolved diffraction and calculation can be combined to substantiate an interpretation. A good example is the electron diffraction result of Ihee, Cao and Zewail [199], that the primary product of Fe(CO)_5_ dissociation is Fe(CO)_4_ in its S_0_ state, not in its ground state (T_1_); the conclusion was based on the calculated angles and distances and was stimulated by our suggested singlet path (Section 7).

These interpretations assume that the wave packet moves in a coherent way, without statistical redistribution of energy (without thermalization) over a time of at least a few picoseconds. This assumption is supported by the coherent oscillations observed over this time range. Nevertheless, energy must flow out from the “active” mode in ultrashort time in some coherent manner and non‐exponentially; for example, energy from the twist mode of ethylene must be transferred elsewhere so that the molecule can reach its perpendicular minimum in 20 fs (Section 4). (The idea is further explained, also with more examples, in [83]. Theoretical examination seems desirable, however).

Furthermore, consistent with the absence of thermalization, the wave packet seems to remember over some time, where it came from. This idea is a prerequisite of the suggested interpretations of wavelength dependences. It seems also to be worth theoretical examination. It is widespread to assume that the molecule first relaxes down to S_1_, from where the reaction then takes place. But in the absence of a memory, this mechanism would not allow for a dependence on the initial state.

This memory can also be described as conservation of momentum direction. Such a rule requires that the potential is sufficiently flat transversely to the propagation. Apparently, this is often the case. On the other hand, there will be coordinates, along which the potential is not flat, so that a valley may be formed that can guide the wave packet. If this valley exhibits a slight curvature, part of the kinetic energy will be redirected. This may be a mechanism of the coherent redistribution of energy [83] (without equipartion or thermalization), mentioned above.

Interesting variants are offered by the metal carbonyls (Section 7): From a bound higher excited state, Fe(CO)_5_ is suggested to first go down to the repulsive S_1_, although not vertically. (A vertical process would leave the Fe–C–O angle untouched at 180°, whereas bending of this group before dissociation was required to explain the rotation of the expelled CO). On the other hand, Cr(CO)_6_ and heavier homologs seem to pass from a higher bound S_*n*_ directly to a repulsive potential, without any sign that lower bound states are involved.

All this can be understood as phenomena of coherent photochemical processes, consistent also with the coherent oscillations that are sometimes observed even in products. (Examples are the coherent oscillations after ring opening of cyclohexadiene in Sections 5.2 and 5.3, those after dissociation of metal carbonyls in Section 7, those observed in the hot ground state of (cis‐ or trans‐) stilbene after relaxation from excited cis‐stilbene [228] or those after cis‐trans isomerization of rhodopsin‐type molecules [229]).

The coherent oscillations are remarkable also in another respect: It means that the wave packet remains compact along all the way down to the product. This in turn means that its path is not distributed over a broad area of the potential and that its diabatic transition to another potential occurs at a localized CoIn, even if an extended IS would be accessible. (Calculated such ISs are compiled by Blancafort [20]). If a wave packet propagates in a harmonic potential, it remains compact. It is surprising that it does not necessarily broaden on its path with repeated turns of direction and passing through the highly anharmonic regions of CoIns. This question certainly deserves some theoretical attention.

Another—perhaps related—question is the suggestion that the wave packet passes diabatically to another potential very suddenly, even if it goes around the lower cone of a CoIn. This was suggested in Section 7 to explain some observations in metal carbonyls. But it obviously also applies for the path around the first CoIn in ethylene or cyclohexadiene.

## Disclaimer

The opinions expressed in this publication are the view of the author(s) and do not necessarily reflect the opinions or views of ChemPhysChem, the Publisher, ChemistryEurope, or the affiliated editors.

## Conflicts of Interest

The author declares no conflicts of interest.

## Data Availability

Data sharing not applicable to this article as no datasets were generated or analyzed during the current study.
